# Bacterial and fungal endophyte communities in healthy and diseased oilseed rape and their potential for biocontrol of *Sclerotinia* and *Phoma* disease

**DOI:** 10.1038/s41598-021-81937-7

**Published:** 2021-02-15

**Authors:** C. S. Schmidt, L. Mrnka, P. Lovecká, T. Frantík, M. Fenclová, K. Demnerová, M. Vosátka

**Affiliations:** 1 Department of Mycorrhizal Symbioses, Institute of Botany, Czech Academy of Sciences, Lesní 322, 25243 Průhonice, Czech Republic; 2 University of Chemistry and Technology, Technická 5, 166 28 Prague 6-Dejvice, Czech Republic

**Keywords:** Agroecology, Biodiversity, Microbial ecology, Plant biotechnology

## Abstract

*Phoma* stem canker (caused by the ascomycetes *Leptosphaeria maculans and Leptosphaeria biglobosa*) is an important disease of oilseed rape. Its effect on endophyte communities in roots and shoots and the potential of endophytes to promote growth and control diseases of oilseed rape (OSR) was investigated. *Phoma* stem canker had a large effect especially on fungal but also on bacterial endophyte communities. Dominant bacterial genera were *Pseudomonas*, followed by *Enterobacter*, *Serratia*, *Stenotrophomonas*, *Bacillus* and *Staphylococcus*. *Achromobacter*, *Pectobacter* and *Sphingobacterium* were isolated only from diseased plants, though in very small numbers. The fungal genera *Cladosporium*, *Botrytis* and *Torula* were dominant in healthy plants whereas *Alternaria*, *Fusarium* and *Basidiomycetes* (*Vishniacozyma*, *Holtermaniella*, *Bjerkandera*/*Thanatephorus*) occurred exclusively in diseased plants. Remarkably, *Leptosphaeria biglobosa* could be isolated in large numbers from shoots of both healthy and diseased plants. Plant growth promoting properties (antioxidative activity, P-solubilisation, production of phytohormones and siderophores) were widespread in OSR endophytes. Although none of the tested bacterial endophytes (*Achromobacter, Enterobacter*, *Pseudomonas*, *Serratia* and *Stenotrophomonas*) promoted growth of oilseed rape under P-limiting conditions or controlled *Phoma* disease on oilseed rape cotyledons, they significantly reduced incidence of *Sclerotinia* disease. In the field, a combined inoculum consisting of *Achromobacter piechaudii*, two pseudomonads and *Stenotrophomonas rhizophila* tendencially increased OSR yield and reduced *Phoma* stem canker.

## Introduction

Oilseed rape (OSR) is a major oil-crop in temperate regions of Europe^1^. Two of the most important diseases of this crop are *Phoma* stem canker disease (syn. blackleg disease) caused by the ascomycetous fungus *Phoma lingam* (asexual form; sexual forms *Leptosphaeria maculans* and *L. biglobosa* of the *L. maculans* complex) and *Sclerotinia* stem rot caused by the ascomycete *Sclerotinia sclerotiorum*. During the growth cycle of winter OSR, fungi of the *L. maculans* complex produce sexual ascospores on colonised stubble in autumn, which infect cotyledons and secondary leaves and cause lesions; from there the pathogen spreads systemically and enters the stem, causing the typical blackleg symptoms in summer^2^. Infection can also spread aerially via asexual pycnospores^3^. *Phoma* stem canker (blackleg disease) causes huge economic losses in OSR^4^. Resistance breeding is often specific and overcome by new virulent races^5^. The causal agent of *Sclerotinia* stem rot, *S. sclerotiorum*, persists in soil as black resting structures called sclerotia, that can either produce soilborne mycelium or airborne ascospores for infection of its host^6^; the wide host range hampers control by crop rotation and development of resistant cultivars is still in its infancy^6^. Thus, extensive application of fungicides is necessary in oilseed rape to control both diseases^6,7^^.^ Oilseed rape does also have high nutrient demands and high doses of (synthetic) fertilisers are required^8^.

Due to the environmental and societal demands to reduce agrochemical inputs, plant beneficial microorganisms are intensively researched as an alternative to synthetic pesticides and fertilisers, also for brassicaceous crops such as oilseed rape^9^. Endophytes promote plant growth by various physiological mechanisms such as increasing nutrient availability for the plant via siderophore production, phosphorus (P) solubilisation and nitrogen (N) fixation, modulating plant growth and stress responses via phytohormone production, and controlling pathogens via production of antimicrobial compounds^10^. Endophytes that inhabit seeds and systemically colonise the growing plant from there lend themselves to an application as seed treatment^11^. Such treatments that remain effective during a longer period of the OSR growth cycle would remove the need for fine-tuned spray timing, which is a pivotal and critical issue in chemical control of *Phoma* stem canker^7^ and *Sclerotinia* stem rot^6^.

In OSR, endophyte communities of culturable bacteria^12,13^ and fungi^14^ have been characterised, with the application as plant growth promoters in contaminated soil^13^, and biocontrol agents^12,14–16^ in mind. While there is an extensive focus on the use of endophytes as biocontrol agents, the potential influence of plant pathogens on endophyte communities has been underexplored so far. Not only can endophytes influence the growth of plant pathogens, but also plant pathogens may change the environment for endophytes^17^ and thus have the potential to alter their community structure. Cultivar resistance against *Verticillium* wilt affected the community structure of bacterial endophytes in oilseed rape^12^. However, to our knowledge, no comparable studies exist for the two other major diseases in OSR, *Sclerotinia* and *Phoma* stem canker.

With the work presented here, we start to close this gap. The two major aims of our study were to assess the effect of *Phoma* stem canker infection on bacterial and fungal endophyte communities in oilseed rape and to explore their potential for plant growth promotion and biocontrol. Similar to the previous studies cited above, we focused on culturable endophytes for potential industrial production as beneficial inocula. Isolates were characterised in vitro for their plant growth promoting properties. A selection of bacterial and fungal isolates representing the major genera was tested *in planta* for plant growth promotion of OSR under nutrient limiting conditions and for control of the two major pathogens *Phoma* stem canker and *Sclerotinia* stem rot. We hypothesised that both bacterial and fungal communities in plants with symptoms of *Phoma* stem canker would differ from communities in healthy plants. We also expected that microbes from the endophyte community in symptomless plants in particular would provide protection to OSR plants from *Phoma* stem canker and/or *Sclerotinia* white rot.

## Results

### Bacterial endophyte communities

**Figure 1:**
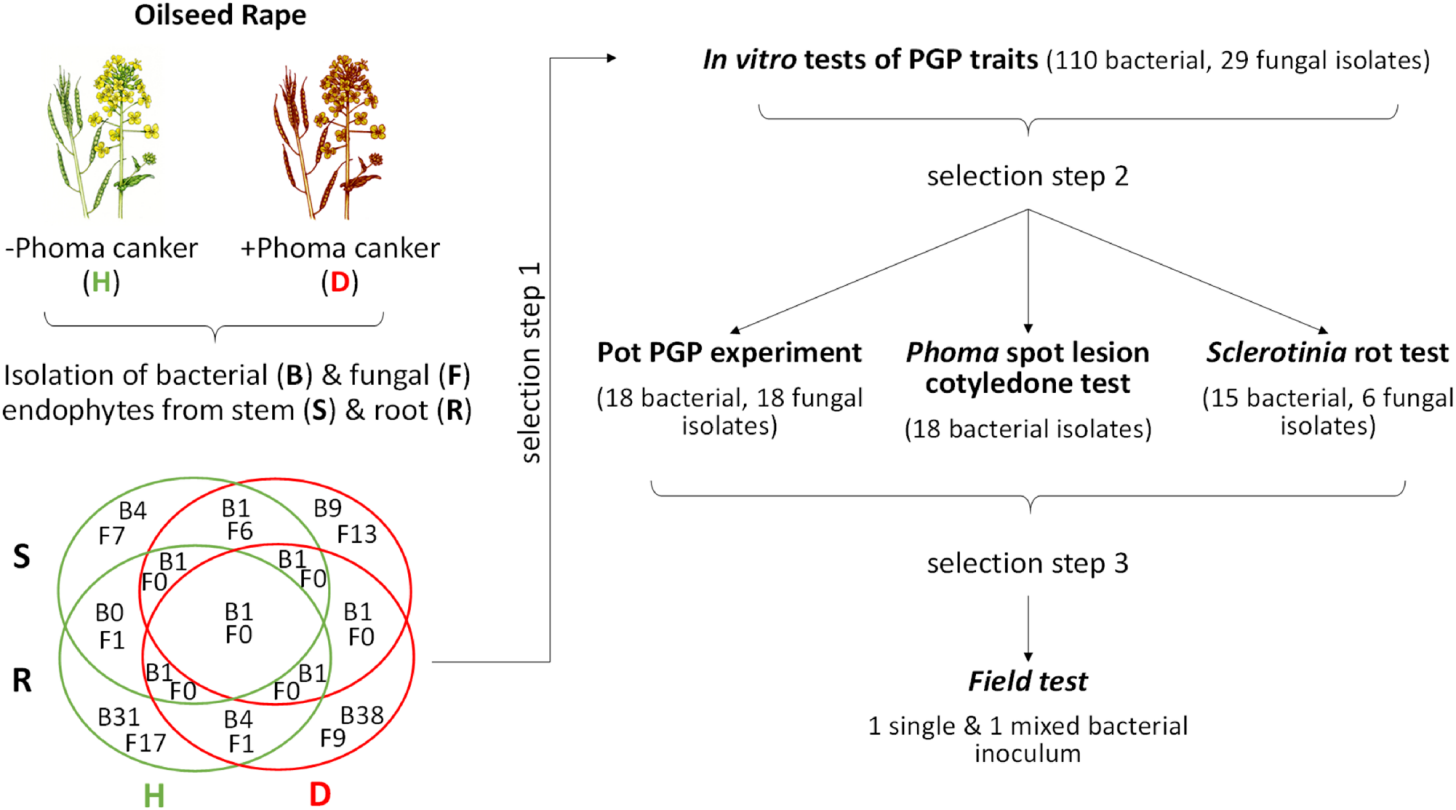
General outline of the study and Venn diagram of the isolated culturable endophytes from symptomless oilseed rape (H, healthy) and oilseed rape showing symptoms of *Phoma* Stem Canker (D, diseased).

**Figure 2:**
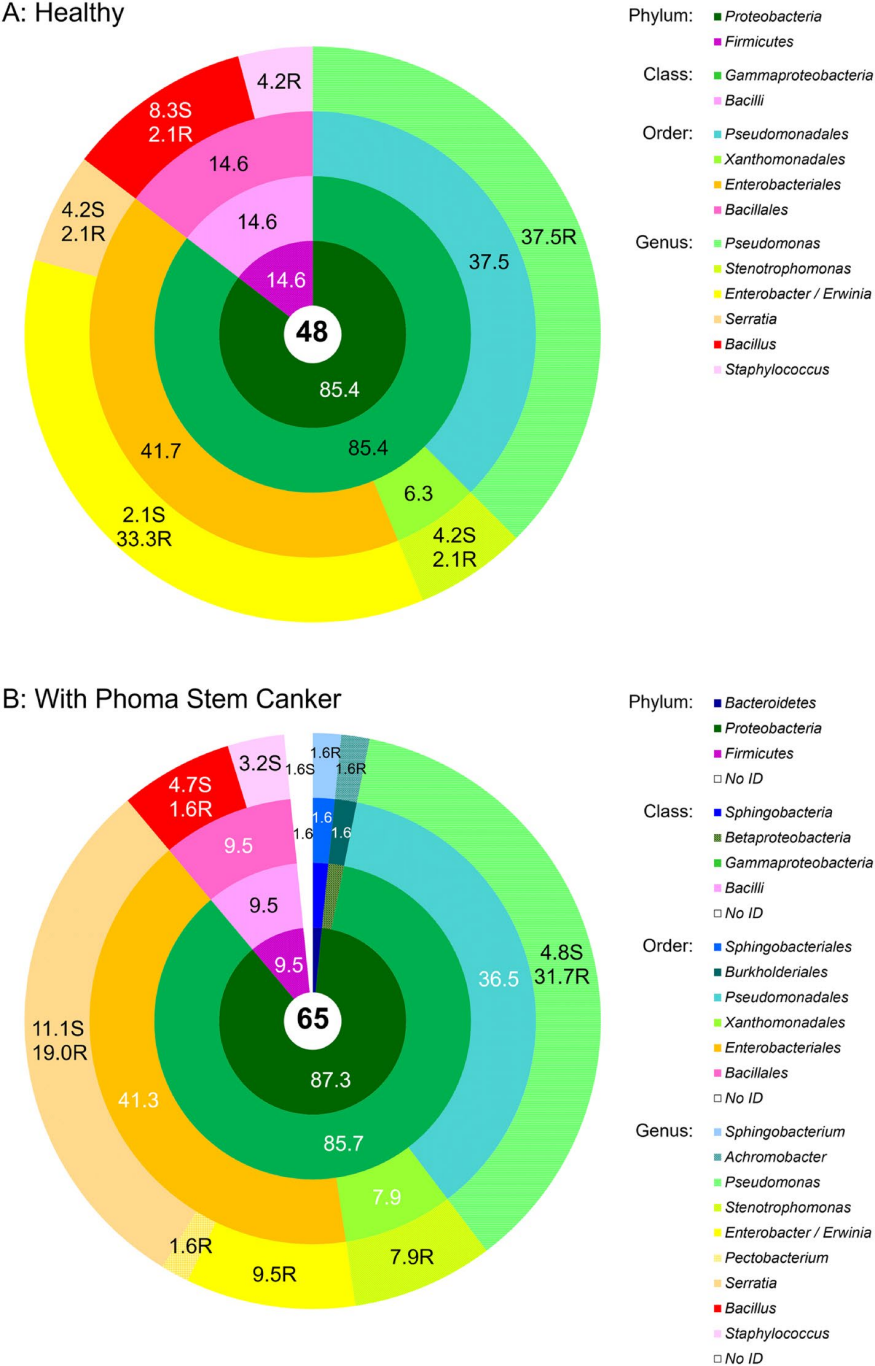
Community composition of the cultivable bacterial endophytic community associated with symptomless oilseed rape **(A)** and oilseed rape showing symptoms of *Phoma* Stem Canker **(B)**. The total number of isolates in each group is shown in the central circle. The chart shows percentages by phyla, classes, orders, and genera progressively from the central to the outermost annuli. N.D. not determined. On the genus level, the abundances of isolates from roots and shoots are presented separately, with the additional letters “R” for roots, and “S” for shoots. Systematic classification according to^49^.

Culturable bacterial endophyte communities of healthy and diseased OSR plants were distinct at the species level (Venn diagram in Fig. 1) but relatively similar at the genus level (Fig. 2A,B). Out of several diversity indices calculated in the study Shannon–Wiener, Gini–Simpson and Pielou evenness indices showed no difference in richness and evenness of bacterial communities isolated from healthy and diseased plants, but Margalef diversity index and Total taxonomic distinctness clearly indicated a higher diversity and total taxonomic breadth of an assemblage in diseased plants (Table 1). This probably arose from several genera (*Achromobacter*, *Pectobacterium* and *Sphingobacterium)* being isolated only from diseased plants, albeit in very low numbers. In both healthy and diseased plants, *Gammaproteobacteria* and *Bacilli* dominated, with the former contributing more than 85% of all isolates (Fig. 2a,b). With a share around 37%, *Pseudomonas* was the prevailing genus in both healthy and diseased OSR. The second largest group were *Enterobacteriales* represented by the genera *Enterobacter* and *Serratia* (Fig. 2b). *Xanthomonadales* with the genus *Stenotrophomonas* were also present in a greater proportion. *Bacilli*, which made up 9.5–15% of isolates, were largely represented by the genus *Bacillus*, and to a lesser extent by the genus *Staphylococcus* (Fig. 2a,b). While the majority of *Pseudomonas* isolates originated from roots, a larger proportion of the *Serratia* and *Bacillus* isolates were isolated from stems (Fig. 2a,b).

**Table 1:** Diversity indices of culturable bacterial and fungal communities in healthy (symptomless) and diseased (with symptoms of *Phoma* stem canker) OSR assessed at the genus level. Total taxonomy distinctness was calculated according to Clarke and Warwick^68^.

	OSR	Diversity indices				
Endophytes	plants	Shannon–Wiener diversity index	Gini–Simpson diversity index	Pielou Evenness index	Margalef diversity coefficient	Total taxonomy distinctness
Bacterial	Diseased	1.70	0.75	0.77	4.45	373
	Healthy	1.45	0.71	0.81	2.97	236
Fungal	Diseased	1.88	0.80	0.86	5.47	378
	Healthy	1.85	0.81	0.84	5.18	303

With MALDI-TOF scores mostly between 2.0 and 2.3, the genus ID of most isolates was certain, whereas the species ID was probable, especially in *Pseudomonas* isolates (Supplementary Table S1). The largest proportion of pseudomonads (around 85% in diseased plants and 50% in healthy plants) belonged to the *P. fluorescens* clade (*P. antarctica*, *P. extremorientalis*, *P. fluorescens*, *P. grimontii*, *P. libanensis*, *P. rhodesiae*, *P. synxantha, P. tolaasi, P. veronii*) within the wider *P. fluorescens* complex^18^. Among members of the *P. fluorescens* clade, *P. tolaasi* was prevalent in the roots of healthy plants, whereas the other species prevailed in diseased plants (Supplementary Table S1). In healthy plants, the *P. corrugata* clade (*P. brassicacearum*, *P. kilonensis, P. thivervalensis*) contributed 50% of *Pseudomonas* isolates. Single isolates of the *P. koreenenis* clade, the *P. pyrolytica* clade and of *P. viridiflava* were isolated from diseased plants (Supplementary Table S1). Remarkably, *P. viridiflava* was only isolated from shoots. Nearly all *Bacillus* isolates were identified as *B. cereus*. Only one *Bacillus* isolate from the roots of diseased plants was identified as *B. mycoides*, another one from diseased shoots as *B. subtilis*. Within other genera, 100% of respective isolates were identified as *Enterobacter amnigenus, Erwinia percinia*, and *Serratia proteamaculans*, respectively (Supplementary Table S1).

### Fungal endophyte communities

**Figure 3:**
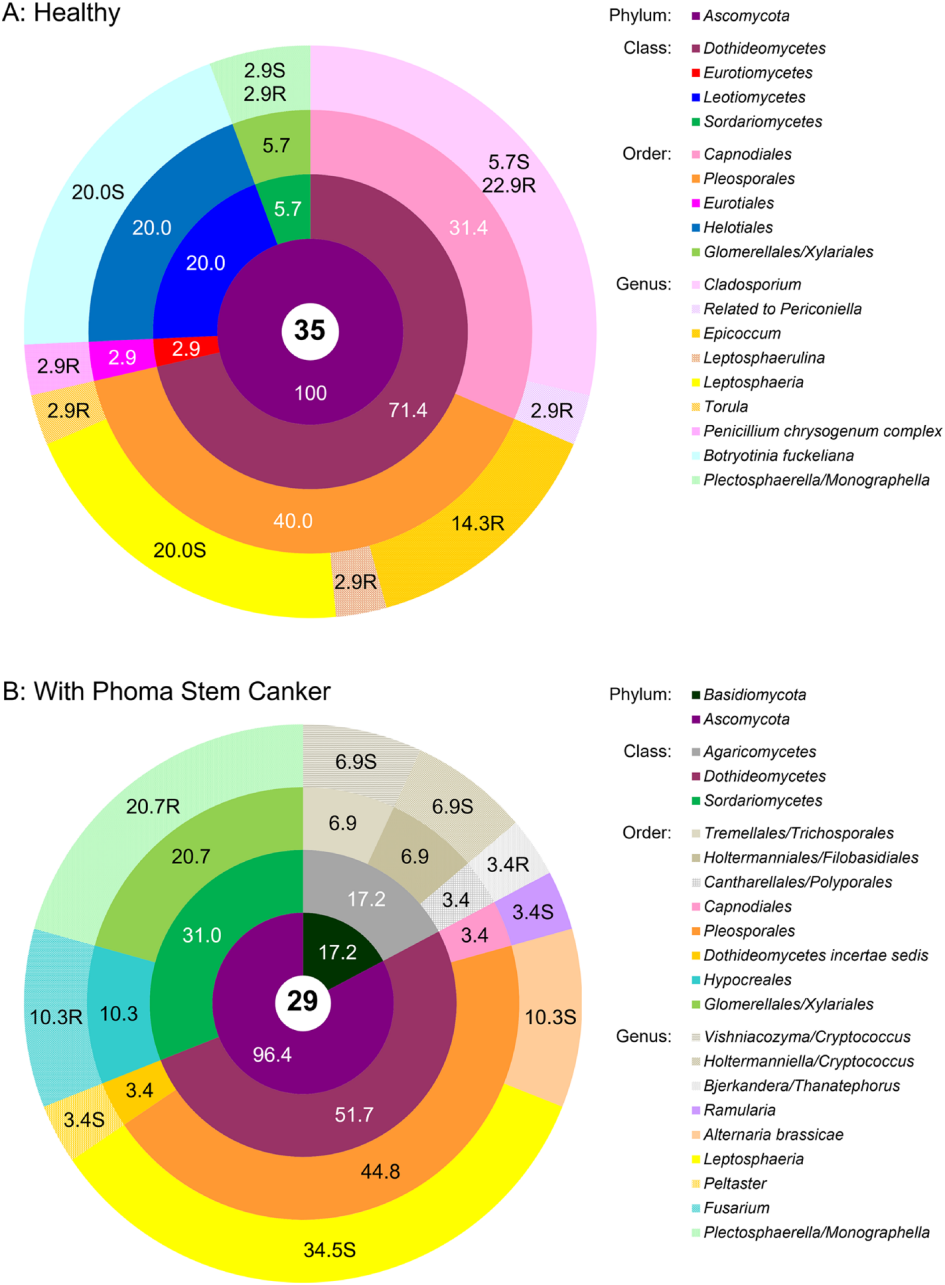
Community composition of the cultivable fungal endophytic community associated with healthy oilseed rape **(a)** and oilseed rape showing symptoms of *Phoma* Stem Canker **(b)**. The total number of isolates in each group is shown in the central circle. The chart shows percentages by phyla, classes, orders, and genera progressively from the central to the outermost annuli. On the genus level, the abundances of isolates from roots and shoots are presented separately, with the additional letters “R” for roots and “S” for shoots. Systematic classification according NCBI Genebank Database.

Diversity indices of culturable fungal communities were similar in healthy and diseased plants (i.e. showing comparable genera richness and evenness) except for the Total taxonomic distinctness index which clearly indicated a broader taxonomic span of endophytic fungal genera in *Phoma* symptomatic plants (Table 1). Most genera had distinct morphotypes and only few (*Cladosporium, Leptosphaeria, Plectosphaerella*) were morphologically variable (Supplementary MS Excel file 1). Nearly all fungal isolates had 99–100% sequence similarity to the closest identified relatives, allowing their taxonomic resolution to the species level (Genbank accession numbers are listed in the Supplementary MS Excel file 1). Contrary to bacterial endophyte communities, fungal endophyte communities were profoundly and qualitatively different in healthy plants and plants affected by *Phoma* stem canker, and taxonomic groups detected in healthy and diseased OSR were mostly represented by different genera (Fig. 3). Only *Ascomycetes* were recovered from healthy plants. In the fungal community isolated from diseased plants, this phylum largely prevailed as well, but 17% of isolates were *Basidiomycetes*, represented by the genera *Vishniacozyma* and *Holtermaniella* (formerly *Cryptococcus*) isolated from shoots, and *Bjerkandera*/*Thanatephorus* (anamorph of the latter: *Rhizoctonia*) isolated from roots (Fig. 3a). Among the *Ascomycetes*, *Dothidiomycetes* were the prevailing group in both diseased and healthy plants; unexpectedly, fungi with 99–100% sequence homology to one causal agent of *Phoma* stem canker (*Leptosphaeria biglobosa*, *L. maculans* species complex) could be isolated in large numbers from the shoots of both diseased and healthy plants (20% and 34.5% of isolates, respectively). The only other genus that could be found in both healthy and diseased plants was *Plectosphaerella* (*Sordariomycetes*, *Glomerellales*). Isolates of this genus were morphologically diverse (see Supplementary MS Excel File 1). While the large majority of closely related sequences in GenBank is classified as *Plectosphaerella*, identification by intergenic spacer sequencing does not allow a clear differentiation from *Monographella* (*Sordariomycetes*, *Xylariales*, 99–100% sequence homology to members of both genera). All other fungal genera were exclusively isolated from either healthy or diseased plants. In healthy plants, *Cladosporium* (*Capnodiales*) was a dominating Dothideomycete (29% of fungal isolations) that was isolated from shoots as well as from roots (Fig. 3a). Isolates of this genus also exhibited significant morphological diversity (Supplementary MS Excel file 1). *Epicoccum* was also isolated in relatively large numbers in healthy plants; other species of *Dothideomycetes* detected were *Leptosphaerulina*, *Torula* and isolates related to *Periconiella* (94% sequence homology). In diseased plants, isolated *Dothideomycetes* belonged to the genera *Alternaria*, *Ramularia* and *Peltaster* (Fig. 3b). Among other Ascomycete classes, *Botrytis* (*Helotiales*) was only found in healthy plants whereas *Fusarium* (*Sordariomycetes*) was only isolated from diseased plants. While they cannot be distinguished from *Sclerotinia* by their intergenic spacer region sequence, all *Botrytis* isolates could be identified morphologically by their typical sporulation.

### Plant growth promoting traits of bacterial and fungal endophytes determined in vitro

Siderophore production and the ability to solubilise P was particularly widespread among *Serratia* isolates and *Pseudomonas*, especially those of the *P. fluorescens*-clade, but less frequent among isolates of the genera *Stenotrophomonas*, *Enterobacter*/*Erwinia* and *Bacillus* (Supplementary Table S1). All tested bacteria and fungi had antioxidative activity (Supplementary Tables S1 and S2). Phytohormone production was also common among bacterial and fungal endophytes. Production of the auxin IAA was generally higher than production of cytokinins (iP, IPR) in both bacteria and fungi. IAA-production of fungi was generally at least one order of magnitude higher than that of bacteria. Bacteria mostly produced iP but no iPR (Supplementary Table S1), whereas iPR was prevailing in fungi among the cytokinin compounds measured (Supplementary Table S2). High cytokinin production was particularly found among isolates of the genera *Stenotrophomonas*, *Bacillus* and *Cladosporium*. Only two of all tested fungal isolates (*Penicillium chrysogenum* RHR13 and *Epicoccum* sp. RHR15) produced very high levels of the mycotoxins roquefortin C and meleagrin. While *P. chrysogenum* is well known to produce both of these mycotoxins^69^, the production of either roquefortine C or meleagrin by *Epicoccum* sp. has yet not been reported to our knowledge.

**Figure 4:**
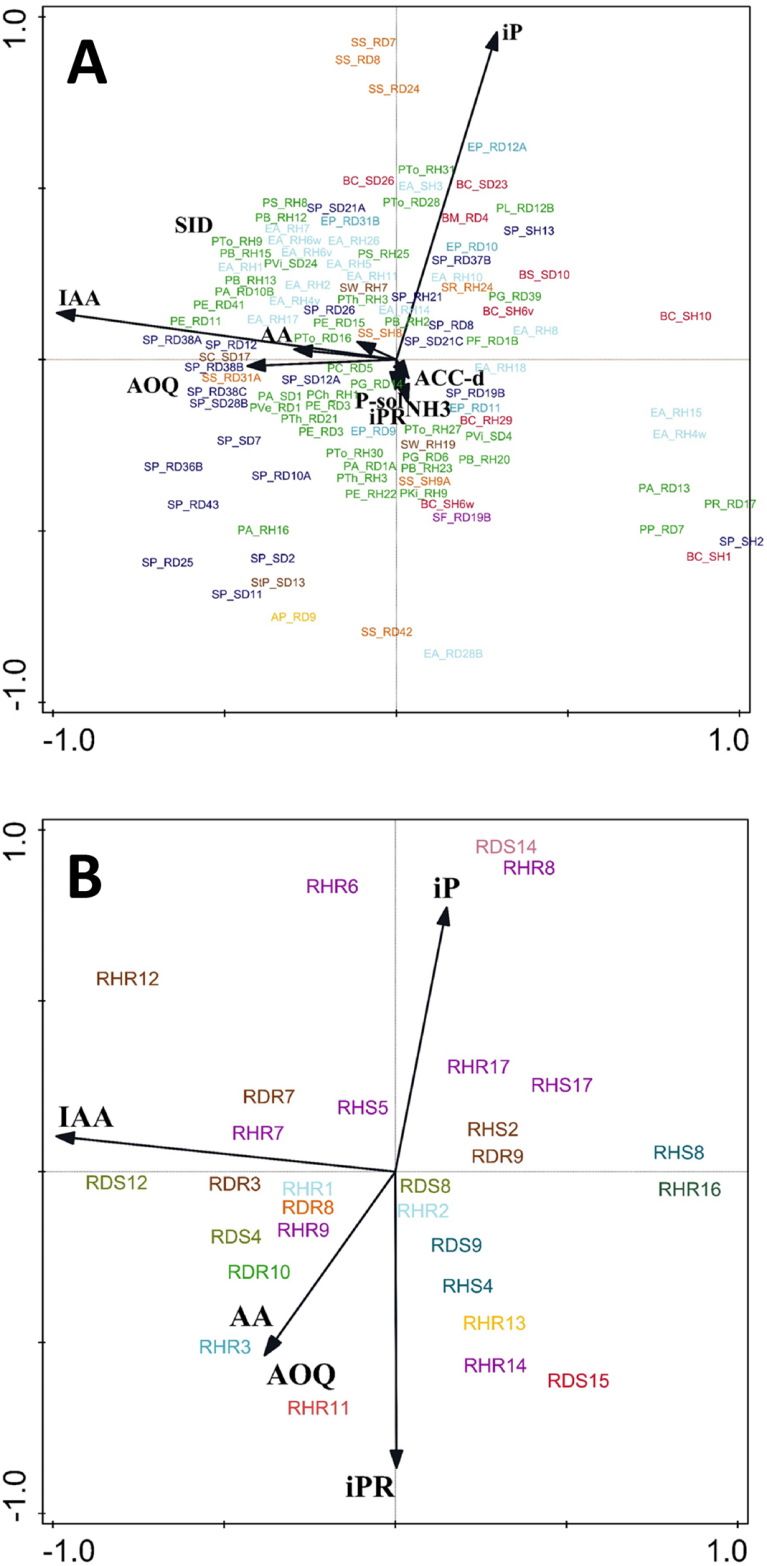
Ordination PCA diagram of bacterial **(A)** and fungal **(B)** isolates. First two axes explained 66% **(A)** and 79% **(B)** variation. Colours indicate taxonomic grouping (See Table S1 and S2). The two characters behind the underscore (bacteria) or initial letter “R” (fungi) in the isolates code are related to the origin of the isolate: R, root; S, shoot/stem; H, healthy = without *Phoma* stem canker symptoms; D, diseased = with symptoms of *Phoma* stem canker.

Neither PCA analysis (Fig. 4) nor CCA cluster analysis (Supplementary Fig. S2) based on PGP trait profiles separated genera of endophytic bacteria (Fig. 4A, Fig. S2A) or fungi (Fig. 4B, Fig. S2B) into distinctive clusters. The origin of isolates (shoot vs. root, diseased vs. healthy) had no influence on PGP trait profiles of endophytic bacteria and fungi at all (Fig. 4, Supplementary Fig. S2).

Representatives of the bacterial genera *Achromobacter*, *Enterobacter, Pseudomonas*, *Serratia* and *Stenotrophomonas*, exhibiting all or most plant growth promoting traits (importance weighed in the following order: ACC-deaminase > siderophore production = P-solubilisation > IAA > iP = iPR > antioxidative activity AA = AOQ > NH_3_ production) and preferentially isolated from *Phoma* symptomless oilseed rape, were selected for pot experiments with OSR. For the selection of fungal isolates a similar approach was used: weighed importance of the traits: IAA > iP = iPR > antioxidative activity AA = AOQ, preferential origin from *Phoma* symptomless plants, ease of spore production based on our observation and literature records. Representatives of the genera *Cladosporium*, *Epicoccum*, *Leptosphaeria*, *Penicillium*, *Periconiella*, *Plectosphaerella*, *Ramularia*, *Torula* and few isolates from *Tremellales* (related to *Cryptococcus*) were selected for pot experiments with OSR (in the case of *Sclerotinia* rot test only *Cladosporium* isolates were tested).

### Growth promotion of oilseed rape in pot experiments

**Figure 5:**
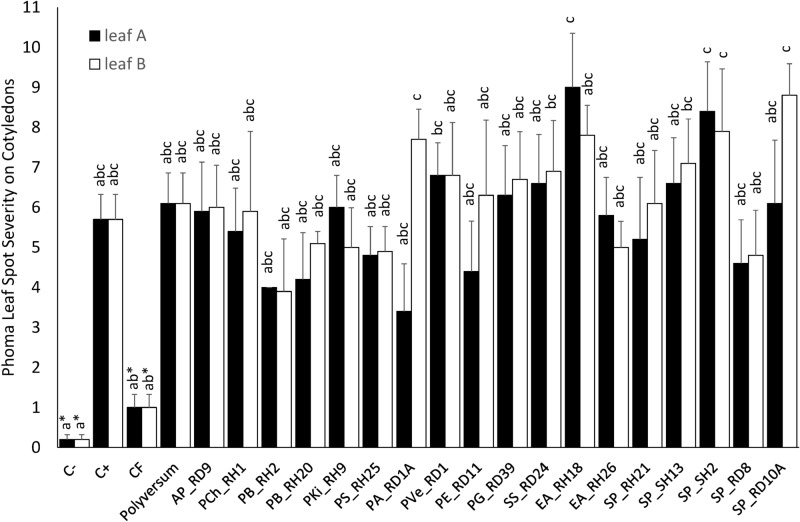
Effect of selected bacterial endophytes on *Phoma* disease (*Leptosphaeria maculans,* anamorph *Phoma lingam*) in a cotyledon assay on oilseed rape seedlings. Data are presented as means ± SE; AP = *Achromobacter piechaudii*, PS =  *Pseudomonas* sp*.*, EA = *Enterobacter amnigenus*, SP = *Serratia proteamaculans, *SS = *Stenotrophomonas* sp.,  C = negative (healthy) control without *Phoma* inoculation and without bacterial inoculation, C + = positive (diseased) control with *Phoma* inoculation and without bacterial inoculation, CF = fungicide treated control, Polyversum = commercial preparation of *Pythium oligandrum.* There was a significant treatment effect on *Phoma* severity in the directly treated cotyledon (leaf A, P = 0.0003) and on *Phoma* symptoms after seed treatment only (leaf B, P < 0.0005). Treatments with the same letters are not significantly different according to Unequal N HSD test. Where Dunnettʼs test showed a significant treatment effect compared to the control C + the significance of this treatment effect is shown as asterisks in superscript.

Although the large majority of selected bacterial strains solubilised P in vitro, none of them promoted the growth of oilseed rape in a P-limited soil significantly (Supplementary Table S3). Red leaf discolouration in all treatments after 102 days of growth except for the control Cp with normal P-fertilisation and a significant increase of growth and pod yield in this control indicated that insufficient P-supply was limiting the growth. Plants were fertilised after 123 days of growth in order to alleviate P-limitation and to detect growth promotion caused by mechanisms other than P-solubilisation, but no significant effects could be found at the end of the experiment. Only tendencies to increase plant dry weight were observed in plants treated with *Achromobacter piechaudii* AP_RD9, *Pseudomonas chloraphis/veronii* PCh_RH1, *Stenotrophomonas* sp. SS_RD24, *Enterobacter amnigenus* EA_RH18 and *Serratia proteamaculans* SP_RH21. Also, cumulative height tended to be increased in plants treated with *Pseudomonas* sp. PG_RD39 (*P. fluorescens* clade), and *E. amnigenus* EA_RH18 (Supplementary Table S3). Isolates *A. piechaudii* AP_RD9, *P. chloraphis/veronii* PCh_RH1, *Pseudomonas* sp. PG_RD39 and *Stenotrophomonas* sp*.* SS_RD24 were selected to be tested as a growth promoting combined inoculum (IG) in a field experiment (see sub-chapter below); *E. amnigenus* EA_RH18 was not included as it showed a tendency to increase *Phoma* severity (Fig. 5). The strains finally included the combined growth promoting inoculum IG did not show any mutual antagonism in vitro (Fig. S3).

None of the tested fungal endophytes increased growth and pod yield of OSR (Supplementary Table S4).

### Control of *Phoma lingam* in planta (cotyledon assay)

In the cotyledon assay, some plants did not emerge or showed non-specific stress symptoms (yellowing and wilting of leaves) and thus could not be included in the analysis. Due to this reduction in the number of replicates (minimum N = 3 as compared to N ≥ 5 in other experiments) and due to a large variation in lesion sizes, only trends were visible in the cotyledon assay. None of the tested bacteria was comparable to the fungicide treatment (Fig. 5). Lesion size tended even to be increased after treatment with some endophytic isolates, most notably with *E. amnigenus* EA_RH18 or *S. proteamaculans* SP_SH2. For *S. proteamaculans* SP_SH2 and some other isolates, this trend was apparent not only in the directly sprayed leaf but also in the non-treated second cotyledon that could have been influenced only by a systemic effect of the seed treatment. Seedlings treated with *Pseudomonas* sp. PA_RD1A (*P. fluorescens* clade) had the lowest lesion score (3.4 vs 5.7 of the non-treated control; Table 1) but only when the leaves were directly sprayed. Generally, there were no positive systemic effects of endophytes when these were applied as seed treatment (Fig. 5).

### Control of *Sclerotinia sclerotiorum* in a pot experiment

**Figure 6:**
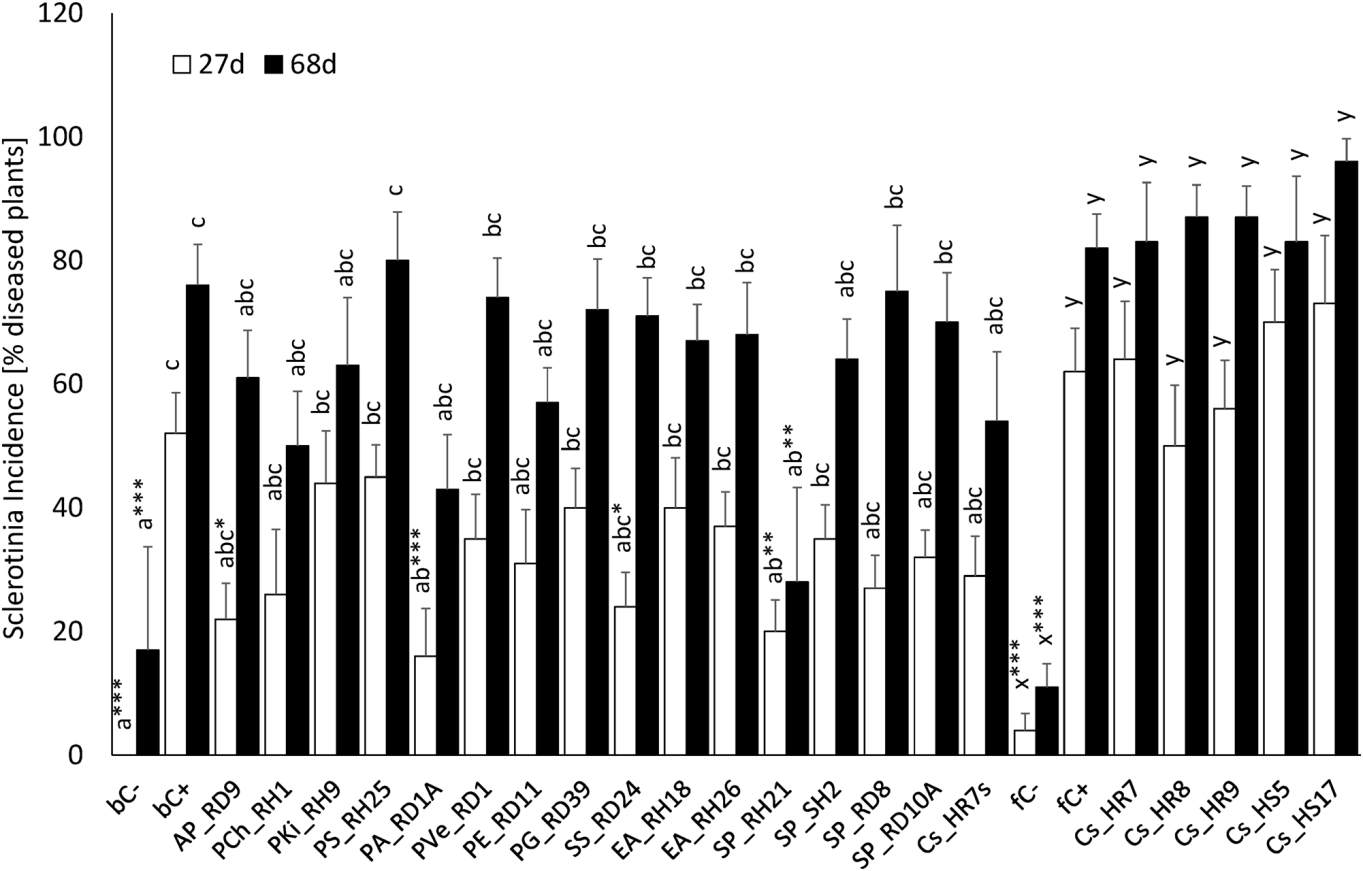
Effect of seed treatment with endophytes on incidence of *Sclerotinia* disease in oilseed rape seedlings. Data are presented as means ± SE; AP, *Achromobacter piechaudii*; Ps.,  *Pseudomonas *sp.; EA, *Enterobacter amnigenus*; SP, *Serratia proteamaculans*; SS, *Stenotrophomonas* sp.; Cs_HR7s, *Cladosporium* sp. HR7 applied as spores; Cs_HR7, *Cladosporium* sp. HR7 applied as mycelium; *bC−,*  negative (healthy) control without *Sclerotinia* inoculation and without bacterial inoculation; *bC+,* positive (pathogen-inoculated) control in *Sclerotinia* infested soil without bacterial inoculation; *fC−,* fungal negative (healthy) control without *Sclerotinia* inoculation and *fC+,* fungal positive control (with pathogen). bC− and bC+ are the corresponding controls for all bacterial treatments and Cs-HR7s, while fC− and fC+ are the corresponding controls for all fungal endophyte treatments applied as mycelium (Cs_). There was a significant treatment effect on incidence of *Sclerotinia* after 27 days and 68 days growth (P < 0.0001). Treatments with the same letters are not significantly different according to Unequal N HSD test (inoculation with cell or spore suspension) or according to Kruskal–Wallis Test. Where Dunnettʼs test showed a significant treatment effect compared to the control C + the significance of this treatment effect is shown as asterisks (* P < 0.05, ** P < 0.01, ***P < 0.001) in superscript.

Amended to soil, *S. sclerotiorum* caused damping off in OSR seedlings. First symptoms were a girdled and discoloured hypocotyl, followed by wilting and finally death of the seedling; symptom development was often accompanied by visible outgrowth of typical white mycelium. The isolates *Pseudomonas* sp. PA_RD1A (*P. fluorescens* clade) and *Serratia. proteamaculans* SP_RH21 significantly reduced incidence (P = 0.001 and P = 0.005, respectively; Fig. 6) after 27 days of growth. *A. piechaudi* AP_RD9 and *Stenotrophomonas* sp. SS_RD24 showed a weaker but still significant effect (P = 0.014 and P = 0.028, respectively). Only *S. proteamaculans* SP_RH21 was able to control *Sclerotinia* significantly (P = 0.002) over a longer period of 68 days (Fig. 6). None of the tested *Cladosporium* isolates had any significant control effect, whether applied as spores (RHR7), or as mycelial inoculum. *Pseudomonas* sp. PA_RD1A had a negative effect on OSR seedlings emergence even before visible symptoms of *Sclerotinia* infection became apparent (14 d growth; P = 0,009; Table S5). The most effective isolate *S. proteamaculans* SP_RH21 was selected to be tested as a protective inoculum (IP) in a field experiment.

### Effect of seed treatments with bacterial endophytes on OSR yield, growth and disease incidence under commercial field conditions (Poříčí, Czech Republic)

Except for a highly significant (P = 0.00002 and P = 0.00033, respectively) reduction of stress symptoms (red discolouration of leaf margins) by both IP and IG inocula in early spring (March 2017; Table 2) none of the seed treatments with bacterial endophytes affected growth, yield and diseases of OSR significantly under commercial field conditions. Yet, some trends were observable. Despite its efficacy against *Sclerotinia* in the pot experiment *S. proteamaculans* SP_RH21 (treatment IP) failed to control the disease in the field (Table 2). Albeit not statistically significant, there was a trend towards increased yield (+ 15%) and reduced severity of *Phoma* stem canker (-42%) after seed treatment with the IG consortium consisting of *A. piechaudii* AP_RD9, *Pseudomonas* sp. PCh_RH1, and PG_RD39, and *Stenotrophomonas* sp. SS_RD24. Fungicide treatment significantly lowered disease incidence of *Sclerotinia* stem rot and *Alternaria* on stems (Table 2).

**Table 2:** Effect of bacterial endophytes applied as a seed coat on winter oilseed rape yield, Disease incidence of *Phoma* stem canker (*Phoma lingam*), *Sclerotinia* stem rot (*Sclerotinia sclerotiorum*), and *Alternaria* stem infection and on oilseed rape overwintering under commercial field conditions (Poříčí, Czech Republic).

Var	Yield	Yield in-crease (%)	Disease incidence Aug 2017, BBCH81–89 (%)			Overwintering March 2017		
	Yield (t/ha)		*Phoma* stem canker	*Sclero-tinia* stem rot	*Alter-naria* on stems	Plants per m^−2^	Plants with *Phoma* lesions (%)	Red discolouration of leaves (%)
C	2.5 ± 0.23 a	100.0	26 ± 3 a	42 ± 4 a	42 ± 4 a	29 ± 1.4 a	9 ± 3.6 a	18 ± 3.7 b
IP	2.7 ± 0.29 a	105.4	24 ± 3 a	48 ± 4 a	49 ± 4 a	31 ± 1.8 a	6 ± 2.2 a	8 ± 2.0 a^(P=0.00002)^
IG	2.9 ± 0.28 a	115.3	15 ± 3 a	57 ± 4 a	57 ± 4 a	33 ± 1.8 a	9 ± 2.7 a	11 ± 2.2 a^(P=0.0033)^
− F	2.5 ± 0.20 a	100.0	22 ± 2 a	56 ± 3 b	55 ± 3 b	–	–	–
+ F	3.0 ± 0.15 a	117.2	21 ± 3 a	41 ± 3 a	42 ± 3 a	–	–	–

Data are presented as means ± SE. C = Control; IP = *Serratia proteamaculans* SP_RH21; IG = combined inoculum of *Achromobacter piechaudii* AP_RD9, *Pseudomonas chloraphis/veronii* PCh_RH1, *P. grimontii/fluorescens* PG_RD39 (*P. fluorescens* clade), *Stenotrophomonas* sp. SS_RD24; − F = no fungicide treatment + F = fungicide treatment in spring. Where Dunnett test showed a significant treatment effect compared to the control C + the significance of this treatment effect is shown as P values in superscript brackets. Different letters indicate significant differences in nested factorial ANOVA (fungicide treatment) or Tukey HSD test nested within strip (bacterial seed treatment).

## Discussion

In most studies on microbial communities that rely on cultivation^12,14^ the number of isolated strains is limited due to the time effort needed for isolation and isolate characterisation. The small sample size of 48/65 bacterial isolates and 35/29 fungal isolates from healthy/diseased plants, respectively, in this study means that the relative abundance of bacterial species needs to exceed 5–6% and the relative abundance of fungal species needs to be greater than 8–10% to be detected with 95% probability^19^. Thus, only dominant genera were securely detected in our study. Small sample size means a further risk that observed differences, especially quantitative shifts, might be based on stochastic events^13^. This was especially apparent for bacteria, where the considerable difference in Margalef index and Total taxonomic distinctness may be due to genera detected in very low numbers (*Achromobacter*, *Pectobacter* and *Sphingobacterium*). In addition, differences in bacterial communities manifested themselves mainly at the species level, where identification via MALDI-TOP was uncertain but only probable for most isolates. On the contrary, fungal communities in healthy and diseases plants differed not only quantitatively but also qualitatively in their dominant groups. Such profound differences, as also observed in poplar under contrasting environmental conditions^20^, appear unlikely to be due to stochastic variation alone, but indicate a strong effect of plant disease on endophyte communities. Though leaves were sampled in one of the symptomless plants next to stems while only stems were sampled in diseased plants it is improbable that this difference had a significant influence on the composition of microbial communities in this study. Neither bacterial nor fungal communities isolated from the aboveground parts of healthy plants were richer in isolated taxa compared to diseased plants as would be expected from the inclusion of another organ (leaves). Furthermore, the differences in fungal genera between diseased and healthy plants were also profound in the roots and not only in the aboveground plant parts.

Unexpectedly, isolates with 99–100% sequence homology to the plant pathogen *Leptoshaeria biglobosa* could be isolated in high frequency from both apparently healthy plants as well as plants with blackleg symptoms. As a causal agent of *Phoma* stem canker, *L. biglobosa* has been found to prevail over *L. maculans* in Eastern European countries^21^ and causes major damage in this region, especially in later growth stages during summer (also the time of our sampling)^22^. Previously it has been considered less aggressive than *L. maculans*, albeit also dependent on external conditions^23^. Its isolation from apparently healthy OSR plants is an indication that it also can be present asymptotically in OSR. Some isolates of the species may even promote OSR growth^14^. Moreover, strong interspecies sequence homology makes secure identification of the species not possible based on intergenic spacer sequences alone. There is also strong possibility that several *Leptosphaeria* species were present in our samples as *Leptoshaeria* isolates recovered in this study showed considerable morphological diversity when grown on MEA Agar (Supplementary Excel file 1). Interestingly, two other sequences with 99–100% sequence homology to our isolates were described as stemming from *Pithomyces*, a *Phoma* isolate causing leaf spot in Japanese horseradish (KR998327.1 and KR998328.1, unpublished).

The other fungal group that could be isolated from both asymptotic and symptom-bearing plants plants in this study, *Plectosphaerella*/*Monographella,* was previously isolated from the rhizosphere of OSR^16,24^. While a majority of closest related sequences in Genbank were characterised as *Plectosphaerella*, a large number is described as *Monographella*^25^. Although these two genera belong to different orders of *Ascomycetes* (*Glomerellales* vs. *Xylariales*), intergenic spacer and ribosomal sequences show nearly 100% homology, indicating that their taxonomy still needs to be resolved.

Of the fungal isolates exclusively originating from either plants with blackleg symptoms or symptomless plants, a larger proportion were (potentially) plant pathogenic species, i.e. *Botrytis*, *Fusarium* and *Alternaria*. The observation that *Botrytis* (as well as *Cladosporium* and *Epicoccum*) were isolated in larger numbers, but only from symptomless plants indicates their potential suppression in diseased plants. *Leptosphaeria maculans* increases the level of some glucosinolates in OSR^17^. Even if this does not affect the pathogen itself, it may inhibit other fungi. Other examples of mutual suppression have been found among the endophytic fungal community of OSR where, among other (potential) plant pathogens, *L. biglobosa* inhibited *S. sclerotiorum*, an ascomycete closely related to *Botrytis cinerea *^14^. Conversely, *Alternaria* and *Fusarium*, (as well as *Basidiomycetes* of the former *Cryptococcus* group), which were only present in symptom-bearing plants here, might have profited from a change in the micro-environment, such as the release of nutrients in necrotic tissue. *Alternaria*, *Epicoccum*, *Fusarium* and *Penicillium* were also isolated from the endosphere of OSR in China whereas other less frequent species were not^14^. On the other hand, we could not detect *Chaetomium*, *Clonostachys* and *Periconia* which were dominant in their study^14^.

Studies on the culturable endophytic bacterial community of OSR^12,13,26^, including this study, also differed in their outcome, with different genera being predominantly isolated. These differences in outcome may be due to different growth stages (young plants vs. flowering vs. post-flowering stage), cultivar choice and environmental conditions (geographical region) and due to differences in sampling size. Not only diseases, as observed here, but also disease resistance (against *Verticillium* wilt) changed bacterial endophyte communities in OSR^12^. We found the endophyte community of OSR being largely comprised of the genera *Pseudomonas*, *Stenotrophomonas*, *Enterobacteriales* (*Serratia* and *Enterobacter*) and *Bacillus* which were also frequently isolated as endophytes of *Brassicaceae* in numerous other studies^9^. This spectrum of bacterial genera is remarkably similar to that of the bacterial community antagonistic to the plant pathogenic fungus *Verticillium dahliae* in the OSR rhizosphere^15,27^, with only small differences. Such similarity of the community of antifungal strains in the rhizosphere and the endophytic flora of OSR might suggest that these genera colonise both compartments and comprise a large proportion of the culturable endophytic community in OSR, but that their antifungal traits are not necessarily expressed *in planta*, as they were equally abundant in healthy plants and in plants displaying blackleg symptoms. Nevertheless, it has to be borne in mind that cultivation-based methods of community profiling favour these three groups over others and cultivation-independent approaches reveal much more diverse endophytic community^28^. This was also the case in OSR, where cultivation independent methods unveiled a much wider range of systematic groups among root-associated bacteria^29,30^ than studies confined to culturable bacteria but other factors come into play here as well, as these authors did not differentiate between the rhizoplane and the endosphere.

Strong P solubilisation and other plant growth promoting traits in vitro did not translate into plant growth promotion *in planta* under P-limiting conditions. Especially the assay with tricalcium phosphate (Pikovskaya’s agar^31^) employed here tends to overestimate the P-solubilising capability of isolates, since most P-complexes in soil are harder to dissolve^32,33^. It is notable, however, that isolates with strong siderophore production and P-solubilising capability detected in vitro showed the strongest trend to increase growth in the pot experiment under P-limiting conditions. They also alleviated stress symptoms in the field and tended to increase yield in the field when applied as combined inoculum. Consortia of plant growth promoting microorganisms have been shown to be more effective than the single strains^34^ probably due to complementarity of their traits and their different ecological optima. This was confirmed for OSR in another study, where growth promotion by single strains of *Pseudomonas*, *Serratia* and *Enterobacter* isolates was only significant when the strains were applied together as one combined inoculum in the field^35^. In a similar way, our consortium consisting of *Achromobacter*, *Pseudomonas* and *Serratia* strains showed a tendency to decrease disease incidence of *Phoma* in the field while the single isolates were not effective against *Phoma* stem canker in the cotyledon assay.

Unexpectedly, isolates arising from symptomless plants were not more effective against fungal pathogens than bacteria isolated from plants with *Phoma* stem canker, and some of the most antagonistic bacteria against *Phoma* and *Sclerotinia* diseases in our *in planta* assays were isolated from diseased plants. Hardly any isolates effective against *P. lingam* could be detected in the cotyledon assay, possibly because the employed method of mycelial inoculation is more invasive and creates greater disease pressure than inoculation with pycnidiospores used by other authors^23,65,66^. We used *in planta* tests with seedlings rather than in vitro assays for the screening of antagonist activity as the latter are not always correlated with *in planta* performance^36^, and do not detect the mechanism of resistance induction, which is particularly common in the genera *Pseudomonas *^37^ and *Serratia*^38^. Bacteria of both genera have been shown to be effective against *Sclerotinia* in OSR previously^39,40^. An apoplastic isolate of *P. viridiflava* effectively supressed growth of three OSR pathogens (*L. maculans*, *S. sclerotiniorum* and *Xanthomonas campestris*) in dual culture-assays^70^. The same isolate also restricted propagation of *X. campestris* and reduced necrotic lesions caused by *S. sclerotiniorum* in OSR leaves and promoted the growth of OSR. The protective effect of the endophyte was probably due to the induction of host resistance via salicylic acid and jasmonic acid signalling pathways and/or production of antimicrobial compounds^70^. Several other bacteria controlled *S. sclerotiniorum* with most of them belonging to the genera *Bacillus* and *Pseudomonas*. Protection mechanisms comprised increased levels of hydrolytic enzymes such as chitinase and β-1,3-glucanase, increased expression of pathogenesis-related proteins and production of antifungal compounds^71,72,74^. While effective plant growth promoters have been identified within the genus *Achromobacter*^42–44^, fewer reports exists on successful biocontrol using this genus^45,46^. In OSR, *Achromobacter* has been found to promote plant growth by improving nutrient uptake^47^. Next to the above mentioned bacteria *Serratia plymuthica* HRO-48 was successfully developed as seed treatment for OSR against *Verticillium dahlia *^11^ and *Phoma lingam*^41^. To our knowledge, the species *Serratia proteamaculans* has not yet been reported as a biocontrol agent in OSR, but an isolate of this species controlled *Alternaria* in tomato^38^.

Our strain of *S. proteamaculans* that was most effective against *Sclerotinia* in the seedling assay failed in the field, possibly because the seedling assay does not mimic natural infection conditions in OSR closely, where the spread via ascospores germinating on senescent petals is the prevailing infection pathway^6^. Systemic colonisation and a long time of survival in the plant is needed if strains applied as seed treatments are to prevent infection via this pathway, where infection occurs in the flowering stage. Alternatively, foliar application is an efficient way of introducing bacteria providing biological control of fungal diseases in oilseed rape but in such a case exact timing of the application within the time window of petal infection by *S. sclerotiniorum* ascospores is crucial^72^. In addition, the imbalanced design of the fungicide application in our field experiment (two out of three control plots but only one out of three IG and IP plots were treated with fungicide) might have putatively lowered the difference in yield and disease incidence between the control and the bacterial treatments IP or IG.

In contrast to a large number of bacterial endophytes providing biological control against OSR diseases the number of efficient fungal strains mentioned in literature is limited to a few, corresponding also to the output of this study where several bacterial strains but no fungal strain exhibited fungal disease suppression. Among fungi, *Gliocladium catenulatum* and *Acremonium alternatum* protected OSR from clubroot caused by *Plasmodiophora brassicae*^73,75^ and *Ulocladium atrum* and *Coniothyrium minitans* reduced incidence and severity of *Sclerotinia* stem rot^76^. However, the profound differences in fungal communities in *Phoma*-symptomless and symptomatic plants found here raise hope that more fungal endophytes antagonistic to fungal diseases might be found.

In conclusion, we have shown considerable differences in fungal and, to a lesser extent, also in bacterial endophyte communities in *P. lingam*-symptomatic and symptomless OSR plants. While the scope of the study could provide only an introductory characterisation of their plant-growth promoting and biocontrol properties, it yielded bacterial strains that are worthy of further study (*Achromobacter piechaudii* AP_RD9, *Pseudomonas chloraphis/veronii* PCh_RH1, *P. grimontii/fluorescens* PG_RD39, and *Stenotrophomonas* sp. SS_RD24). In future experiments, it would be desirable to improve the formulations of the strains^48^, to optimise their application technique and to monitor their long-term survival in the plant.

## Methods

### Isolation of bacterial and fungal endophytes

Endophytes of OSR were isolated by a modified dilution-to-extinction method^50^. OSR plants (cv. ‘Viking’ and ’Catonic’) at growth stage of seed ripening (BBCH > 81^51^) were harvested in mid July 2013 from a research site of SELGEN a.s. near Chlumec n. Cidlinou, Czech Republic (N50:7.7552, E 15:29.3410, 218 m above sea level) at experimental plots not treated with pesticides. In the plots many plants had developed symptoms of *Phoma* stem canker, *Verticillium* wilt, *Alternaria* blight and powdery mildew *(Erysiphe cruciferarum)* while only few plants with *Sclerotinia* stem rot and light leaf spots caused by *Cylindrosporium* sp. were present. We harvested plants with no visible disease symptoms (except for slight presence of *Alternaria* sp. that was ubiquitous) and separately those with symptoms of *Phoma* stem canker. Three plants per variant (symptomless /healthy/, symptomatic /diseased/) were pooled and separated into roots and shoots (shoots consisted of stems only as leaves already deteriorated at this growth stage except of one plant from the symptomless variant where few leaves were present as well). Any adhering soil and debris was removed by washing with tap water. Subsequent isolation of endophytic bacteria and fungi from plant material (shoots and roots separately) was done as previously described^20,52^. Briefly, plant material was surface sterilised for 7 min (2.2% active chlorine with addition of 0.1% Tween 20), transferred to a 70% ethanol solution for 2 min, rinsed four times with sterile deionised water, and cut to small pieces (< 0.5 cm). After thorough mixing of the cut material under sterile conditions approximately 2 g (fresh weight) were homogenised (Ultra-Turrax IKA-T10, max speed, 1 min) in 20 mL of sterile 12.5 mM potassium phosphate buffer (pH 7.1). The homogenate was filtered (polyamide sieve Uhelon 13S, porosity 609 μm), diluted (5 to 50 times for fungi), and plated on 96-well plates with agar media (25 μl medium per well). Fungi were isolated on both synthetic nutrient poor agar (SNA) and nutrient rich malt extract agar (MEA: 2% malt extract, 0.2% meat peptone), each amended with the mixture of antibiotics (50 mg L^−1^ tetracycline, 80 mg L^−1^ streptomycin sulfate, and 60 mg L^−1^ penicillin) to suppress bacterial growth. Twenty-five microliters of the filtered homogenate was applied per well and six plates per given sample and growth medium were used (576 wells in total) to isolate the endophytic fungi. In the case of bacteria, the homogenate was diluted 10^−1^ to 10^−4^ and plated on Petri dishes (diameter 9 cm) with tryptic soy agar (5% TSA, 50% TSA) amended with cycloheximide (100 mg L^−1^). Bacteria were incubated at 20 °C for 48 h while fungi were grown in dark at 20 °C up to 4 weeks. Fungi displaying different morphologies were re-streaked on new plates to obtain clean axenic cultures. Isolated fungi were maintained on SNA and 1:10 strength MEA.

### Identification of bacteria and fungi

Bacterial isolates were identified by MALDI-TOF MS^53,54^ using an Autoflex Speed MALDI-TOF/TOF mass spectrometer and MALDI Biotyper 3.1 software (Bruker Daltonik, Germany). Fungal isolates were morphotyped according to their colony appearance on malt extract agar (MEA; see Supplementary MS Excel file 1). DNA of at least two representatives of each morphotype was isolated^55^, and PCR-amplified for molecular identification based on their ribosomal DNA region^56,57^. Molecular identification and classification was based on the NCBI GenBank database^58^; a similarity of > 97% was used as a threshold for species identification. Higher taxonomic units were selected in the case of lower scoring. When the identity of the two selected representatives differed, all members of the morphogroup were molecularly identified. GenBank accession numbers of submitted fungal sequences are MN275840-MN275887.

### Testing of in-vitro traits of bacteria and fungi

Phosphorus (P) solubilisation, siderophore excretion, ACC-deaminase, antioxidant activity (AA and AOQ = total antioxidative activity expressed in % of quenching activity) and NH_3_ production on peptone were detected as previously described^31,59–62,77^. For the measurement of phytohormones and antioxidants excreted into the culture medium, bacteria were grown in 20 ml LB in 50 ml Falcon tubes for 7 days at 28 °C; fungi were grown in 50 ml Falcon tubes in 15 ml malt extract broth (MEB; 2% malt extract; 0.2% meat peptone; pH 5.5) with 0.5% tryptophan for 10 days at room temperature on a shaker. Auxin indole-3-acetic acid (IAA), the cytokinins N6-(Δ2-isopentenyl) adenine (iP) and N6-(Δ2-isopentenyl) adenosine (iPR) in cultural filtrates were quantified by ultra-high performance liquid chromatography (Acquity UPLC HSS T3 column; 1.8 μm, 2.1 × 100 mm; Waters, Acquity, coupled with tandem mass spectrometry AB Sciex, Qtrap 5500; electrospray ionisation in positive mode). Blank sterile medium served as control^63^. The performance of the endophytic isolates in the in vitro tests was used for the selection step 2 (Fig. 1). The selection was based on following criteria: (1) Number of traits where the particular strain scored high; importance of the traits was weighed according the following order: ACC-deaminase > siderophore production = P-solubilization > IAA > iP = iPR > AA = AOQ > NH_3_ production for bacteria, IAA > iP = iPR > AA = AOQ for fungi, (2) Origin (we preferred isolates from symptomless plants over isolates from symptomatic plants), (3) Taxonomic placement of the isolates (we aimed at covering a wide taxonomic diversity in order to arrive to a broad group of strains putatively complementing each other in function and ecophysiological demands).

### Mycotoxin assessment

Fungal isolates were grown on MEA enriched with 2% oilseed rape extract at 21 °C. Mycelial growth was quantitatively transferred into 250 ml Erlenmeyer flasks, mixed with 40 ml extraction solvent (acetonitrile : 1% formic acid in water, 50:50), homogenised using ultraturrax and shaken for 60 min. The supernatant was filtered into a 50 ml volumetric flask and filled up to the line with the extraction solvent (same as specified above). This extract was then analysed according the methodology previously published for quantitative determination of 57 mycotoxins (including *Fusarium* and *Alternaria* mycotoxins, ergot alkaloids, aflatoxins, fumonisins, and others)^64^. For separation and detection of the analytes, combination of ultra-high performance liquid chromatography (machine equipped with an Acquity UPLC HSS T3 column; 1.8 μm, 2.1 × 100 mm; Waters, Acquity), coupled with tandem mass spectrometry (AB Sciex, Qtrap 5500; electrospray ionisation in positive and negative mode) was used. For the quantification purposes, matrix-matched calibration standards of the mycotoxins (blanks, 0.05–1000 ng ml^−1^) were prepared using blank medium extracted by the procedure described for the samples.

### In planta testing for plant growth promotion

Pots were filled with 1.2 L of autoclaved substrate (7:3 autoclaved zeolite:sand mixture v:v; bulk density 1.17 g ml^−1^) amended with 11 g L^−1^ apatite resulting in a dose of 1200 mg insoluble P per pot. Fertiliser (Osmocote 11:10:18 nitrogen (N):phosphorus (P):potassium (K); 5–6 month release; Everris, The Netherlands) was added to the substrate (1.2 g per pot) resulting in a concentration of 50 mg available P, 130 mg N and 190 mg K per pot; a high P treatment received 5 g Osmocote per pot (260 mg P per pot). Pre-selected bacterial endophytic isolates based on the in vitro test performance were grown in Luria Bertani (LB) broth for 2 days. Seeds of oilseed rape ʻSherlockʼ were surface sterilised (30 s in 70% Ethanol, 15 min in 4.7 NaOCl, 3 × washing) and soaked in cell suspensions of the endophytic bacteria (re-suspended in 0.01 M MgSO_4_, target OD_600nm_ = 0.5 resulting in 10^8^–10^9^ CFU ml^−1^); seeds for controls were soaked in sterile MgSO_4_. Four treated seeds were sown per pot and thinned to one plant per pot after emergence. Pre-selected fungal endophyte isolates based on the in vitro test performance were grown on filter paper laid on MEA and seeds were allowed to germinate on the mycelial mat and then planted into pots; seeds for controls were germinated on sterile filter paper laid on MEA. After 123 days of growth, plants were fertilised with a mixture of Kristalon fertiliser (AGRO CS a.s., Czech Republic) for tomatoes (N:P_2_O_5_:K_2_O 7.5:12:36 + 4.5 MgO + 10 SO_3_ + Trace elements (B Cu, Fe, Mn, Mo, Zn) and KH_2_PO_4_ dissolved in water (1.67 g Kristalon + 0.17 g KH_2_PO_4_ per L, pH 6.1). Four-hundred ml of this solution were added per pot, resulting in a final concentration of 0.05 g P, 0.05 g N and 0.22 g K (ratio 1:1:4.4), 0.018 g Mg and 0.027 g S per pot. Plants were harvested after approx. 190 days of growth and a number of growth parameters assessed (number of shoots and pods, maximum shoot height, cumulative shoot height, shoot, pot and root dry weight/all dry weights at 65 °C/together with the phenological phase/BBCH scale/).

### In planta test for activity against *Phoma* leaf spot (*Leptosphaeria maculans*)

Antagonistic activity of the pre-selected bacterial endophytes against *Phoma* leaf spot was tested with a cotyledon assay on OSR seedlings^23,65^. Twelve mycelial plugs of *Leptosphaeria maculans* isolate ICBN3 (provided by Dr. Koopmann, University of Göttingen, Germany) grown on V8-agar were used to inoculate 100 ml liquid cultures. Culture medium was prepared as follows: 20 g L^−1^ oilseed rape leaves and roots were shredded in a mixer, boiled for 5 min, filtered, amended with 2% malt extract and 0.2% meat peptone, portioned into 300 ml flasks, amended with 3 mm filter paper pieces, and autoclaved; fungal cultures were grown for 3 weeks at 20 °C at 200 rpm before the overgrown filter paper pieces were retrieved for inoculation. Surface sterilised seeds of oilseed rape ʻSherlockʼ were treated with cell suspensions of the bacterial endophytes as described above and sown in 50 ml plastic falcon tubes filled with an autoclaved vermiculite:zeolite mixture amended with 0.15 g osmocote (NPK 12-11-17 + 2 MgO + trace elements fast release within 6 weeks); 2 ml of bacterial cell suspension were added to the substrate. After emergence, plants were thinned to 1–2 seedlings per tube and secondary leaves of plants were regularly removed. After full development of cotyledons (19 days plant age), one cotyledon was punctured with a needle on each half and the wounds treated with 10 µl of bacterial endophyte cell suspensions (resuspended in 0.01 M MgSO_4_, 10^8^—10^9^ CFU ml^−1^) to test a direct protective effect. The other cotyledon was left untreated to test for the systemic effect of endophytes (introduced via seed treatment). Additional controls were set up with a commercial fungicide (Horizon 250EW, Bayer Crop Science, active ingredient tebuconazole) and a commercial biocontrol agent (Polyversum; Biopreparáty Spol. s r. o., Czech Republic; active ingredient *Pythium oligandrum*) which were prepared according to manufacturer’s instructions and applied to wounded cotyledons. Two days later (21 days plant age) all cotyledons were punctured on each side with a needle and covered with filter papers overgrown with mycelium of *Leptosphaeria maculans* (non-inoculated controls C- were covered with sterile filter papers). Filter papers were attached to leaves with a drop of wax. Inoculated plants were kept in a moist chamber at 20 °C for two days and then grown in a growth chamber (16 h/8 h light/dark cycle, 21–23 °C) for further 14 days before lesion sizes were assessed (scale according Chèvre et al.^66^, modified: 0 = no lesion, 1 = lesion size 1.5 mm, 2 = lesion size 1.5 mm and chlorosis, 3 = lesion size 1.5–3 mm, 4 = lesion size 1.5–3 mm, chlorosis, 5 = lesion size 3.1–5 mm, 6 = lesion size 3.1–5 mm, chlorosis, 7 = lesion size 3.1–5 mm, severe chlorosis, 8 = lesion size > 5 mm, 9 = lesion size > 5 mm, chlorosis, 10 = lesion size > 5 cm, severe chlorosis, 11 10 = lesion size > 5 cm, severe chlorosis, withered or sporulation).

### In planta testing for activity against *Sclerotinia*

*Sclerotinia sclerotiorum* strain DBM 4244 (Collection of Yeasts and Industrial Microorganisms, Institute of Chemical Technology, Prague, Czech Republic) was inoculated onto OSR and re-isolated from diseased plants prior use. The experiment was set up in 200 ml pots filled with an autoclaved mixture of horticultural, peat-based substrate and sand (80:20, v:v) This autoclaved substrate was inoculated with *S. sclerotiorum* grown for 4 weeks on autoclaved millet (0.75% m:v) and pre-incubated in tightly closed plastic bags for further 4 weeks according El-Tarabily et al.^67^, modified). Bacterial endophytes were applied on surface-sterilised seeds as described above (~ 10^8^ cfu ml^−1^ in 0.1 M MgSO_4_). The fungal strain *Cladosporium* strain RHR7 was applied twice: once as a spore suspension (10^7^ spores ml^−1^ in 0.1 M MgSO_4_ with 10 µl Tween20) and the other time as a mycelial mat on which OSR seed germinated (analogously to other *Cladosporium* isolates). The reason behind was to compare the two methods of introducing the fungus. Seeds of OSR ‘Sherlock’ were soaked for 1–2 h in bacterial or fungal spore suspensions; seeds for controls were soaked in sterile 0.1 M MgSO_4_. *Cladosporium* isolates (RHR7, RHR8, RHR9, RHR14, RHS1, RHS5) were applied by allowing surface-sterilised seeds to germinate on the mycelial mat of an overgrown filter paper placed on MEA, as described above. Nine treated seeds were sown per pot; pots were placed in a warm greenhouse (20–30 °C). Emergence was assessed after 14 days. Additional millet inoculum grown for 14 days at 20 °C (1.6 g per pot) and inoculum grown on autoclaved oilseed rape leaves for 7 days at 20 °C (0.8 g overgrown leaves per pot) was distributed between the plants after 21 days of growth. Concomitantly, pots were moved to a cold greenhouse (T_min_ 8 °C). The percentage of diseased and dead plants (girdled hypocotyl, white mycelial outgrowth at the hypocotyl, wilting) was assessed after 27 and 68 days of growth.

### In vitro test for mutual inhibition of plant growth promising strains

To exclude the risk that PGPR strains (*A. piechaudii* AP_RD9, *Pseudomonas* spp. PCh_RH1, and PG_RD39, and *Stenotrophomonas* sp. SS_RD24) that we planned to apply in a combined inoculum in the field (see below) inhibited each other, potential mutual antagonism was tested in an in-vitro assay as follows. Cells from liquid cultures (grown overnight on tryptic soy medium at 24 °C) were centrifuged and washed once in phosphate buffered saline (PBS). Each of the strains was then diluted in PBS to 0.5 McF (150.10^6^ CFU/ml, OD 0.125 for 550 nm) and evenly spread with a sterile cotton swab on tryptic soy agar (three replicate Petri dishes per strain) and allowed to dry. The other strains were then applied as droplets (5 µl) at a cell density of 5 McF (1500.10^6^ CFU/ml, OD 1.25 for 550 nm) suspended in PBS onto this seeded lawn (3 replicate drops per Petri dish, 9 in total). After a growth period of 72 h at 24 °C, it was checked whether the outgrowing colonies produced clear inhibition zones in the surrounding bacterial lawn.

### Field experiment in Poříčí, Czech Republic

The strains *Achromobacter piechaudii* AP_RD9, *Pseudomonas chloraphis/veronii* (PCh_RH1), *P. grimontii/fluorescens* (PG_RD39, *P. fluorescens* clade), and *Stenotrophomonas* sp. SS_RD24 were applied to OSR seeds as a combined inoculum (IG; for plant growth promotion) and the strain *Serratia proteamaculans* SP_RH21 was applied as a single inoculum (IP; for plant protection) as follows. Cultures growing in LB for 2 days were centrifuged and the cell pellet re-suspended in Ringer solution. For the combined inoculum, optical density at 600 nm of the cell suspensions was measured and pooled in proportions adjusted to a theoretical value of 2.5 for each isolate within the pooled suspension. Cell suspensions were lyophilised (Eco Fuel Laboratories, Prague, Czech Republic) leading to a final concentration of 8 × 10^8^ cells g^−1^ lyophilisate for the combined inoculum IG, and 5 × 10^8^ cells for the single inoculum IP. For incrustation of OSR seeds, these lyophilisates were mixed into an apatite – bentonite carrier (1:1 apatite : bentonite + 1.7% lyophilisate of IG or 0.9% lyophilisate of IP, respectively; all w:w). This incrustation mixture was added to seeds at the ratio 0.05:1 (w:w). The treatment resulted in 4 × 10^4^ CFU per seed (4.26 µg lyophilisate per seed) for the inoculum IG, and 2 × 10^4^ CFU per seed (2.24 µg lyophilisate per seed) for inoculum IP. Treated seeds were sown in a commercial oilseed rape field in Poříčí, Czech Republic (49.8464633 N, 14.6767358 E) in late August 2016 after winter wheat as a pre-crop. An imbalanced, factorial design with the factors bacterial treatment (controls, IP and IG) and fungicide treatment (+ F, − F) was used (Fig. 1). The field was fertilised with 30 t ha^-1^ cattle manure in autumn and with 300 kg ha^−1^ YaraBela Sulfan fertiliser (24% N + 6% S + 7% CaO) and 210 l ha^−1^ DAM 390 in spring. In March 2017, the number of plants per m^2^ and the percentage of plants with *Phoma* leaf spots and with reddish discolouration of leaf margins (indicative of stress) were counted. Three 1 m^2^-squares, spaced evenly across the length of each strip, were assessed per strip. On the 17th of May, + F plots were treated with fungicide (Acanto plus [picoxystrobin, cyproconazole], 1 L ha^−1^). An assessment of the incidence of *Phoma* stem canker, *Sclerotinia* stem rot an *Alternaria* stem symptoms was done on the 7th of July 2017 before the oilseed rape harvest. 60 plants across each strip were assessed for presence or absence of the diseases.

### Statistical analyses

All plant experiments consisted of five replicates per treatment and 10 replicates for the control (non-treated in the experiment on plant growth promotion, C +  = control inoculated with pathogen, in the experiments on disease control) unless stated otherwise. Replicate pots were fully randomised during growth. Acquired data were checked for normal distribution (Shapiro–Wilk´s W test) and homoscedasticity (Levene´s test). and if necessary data were square root or common logarithm transformed before conducting Student’s t-test or Analysis of Variance followed by post-hoc Unequal N HSD test. Significant treatment effects in comparison to the non-treated control were detected using Dunnett’s test. When the assumption of homoscedasticity was not met, non-parametric alternatives (Mann–Whitney-test, Kruskal–Wallis Analysis of Variance) were used to test for significant differences among experimental groups. The field experiment was analysed in March 2017 using a nested factorial ANOVA with the random factor strip nested within seed treatment (factor 1) and fungicide application (factor 2). For disease assessment plants were assigned the number 0 for absence of disease, and 1 for presence of disease, and a nested ANOVA with the random factor strip nested within seed treatment and fungicide application was calculated. All statistical calculations were done with Statistica v. 12 (Dell Inc., Tulsa, USA). Biodiversity indices (Shannon–Wiener Diversity Index, Gini-Simpson's Index, Pielow evenness index, Margalef diversity coefficient and Total taxonomic distinctness^68^) were calculated based on the genera separately for bacteria and fungi.

## Supplementary Information

Supplementary Figure 1.

Supplementary Figure 2a.

Supplementary Figure 2b.

Supplementary Figure 3a.

Supplementary Figure 3b.

Supplementary Information 1.

Supplementary Information 2.
