# Supplementary figures and images for: Bacterial and fungal endophyte communities in healthy and diseased oilseed rape and their potential for biocontrol of Sclerotinia and Phoma disease
Source: Sci Rep. 2021 Feb 15;11:3810. doi: 10.1038/s41598-021-81937-7 (PMC7884388; doi:10.1038/s41598-021-81937-7)

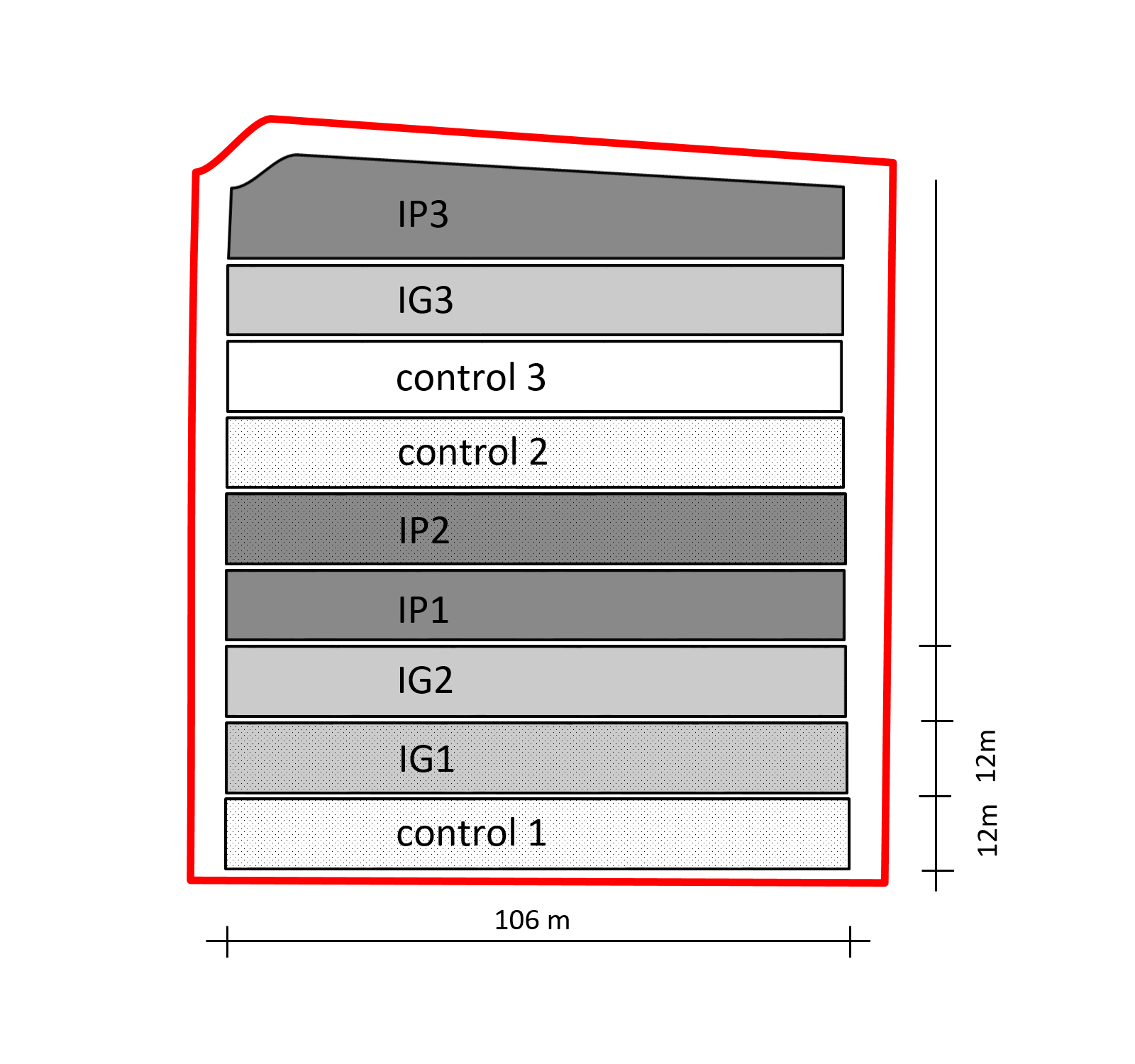

Supplement: Supplementary file 1 — Supplementary Figure 1. [file 41598_2021_81937_MOESM1_ESM.tif]

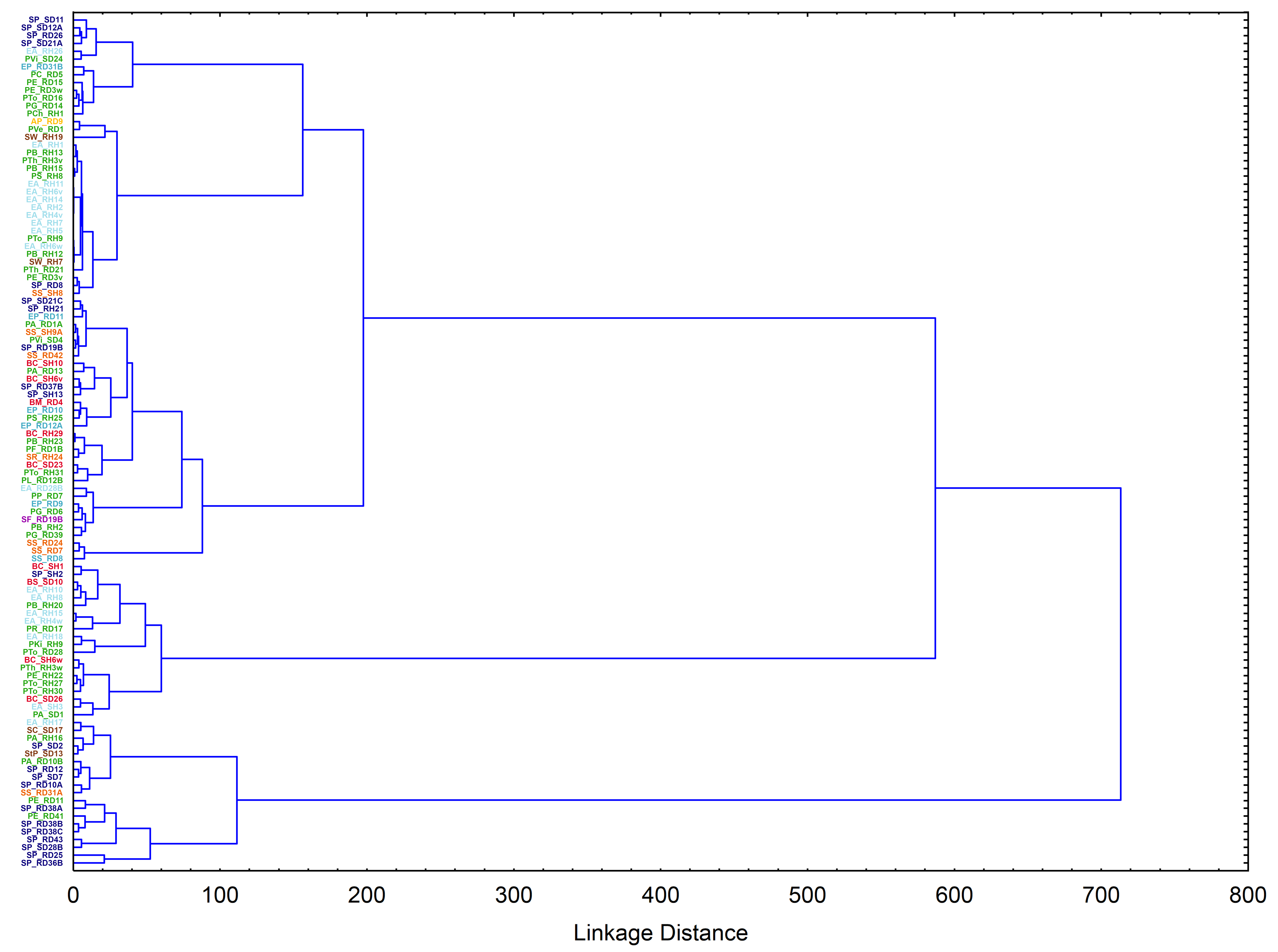

Supplement: Supplementary file 2 — Supplementary Figure 2a. [file 41598_2021_81937_MOESM2_ESM.tif]

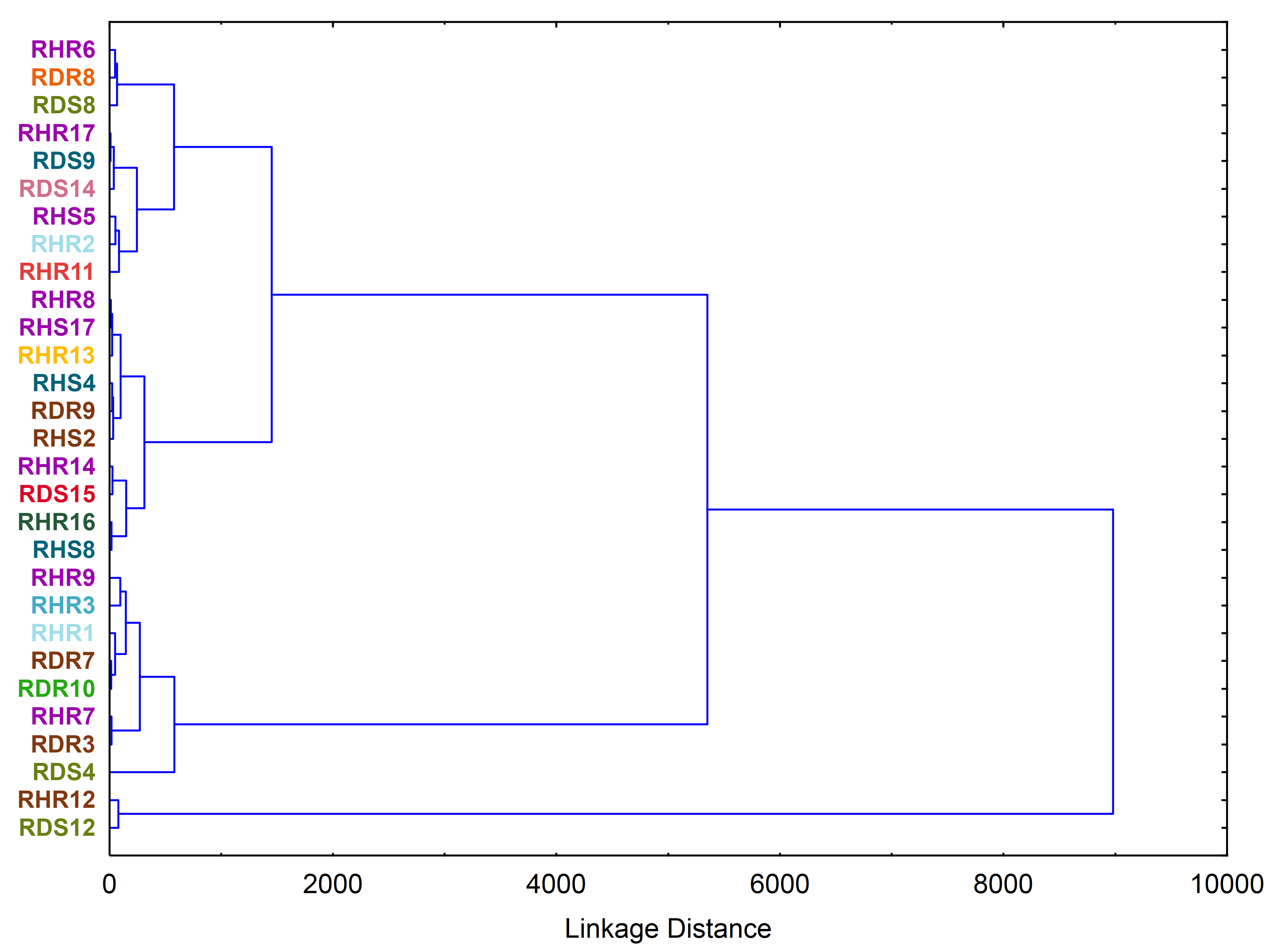

Supplement: Supplementary file 3 — Supplementary Figure 2b. [file 41598_2021_81937_MOESM3_ESM.tif]

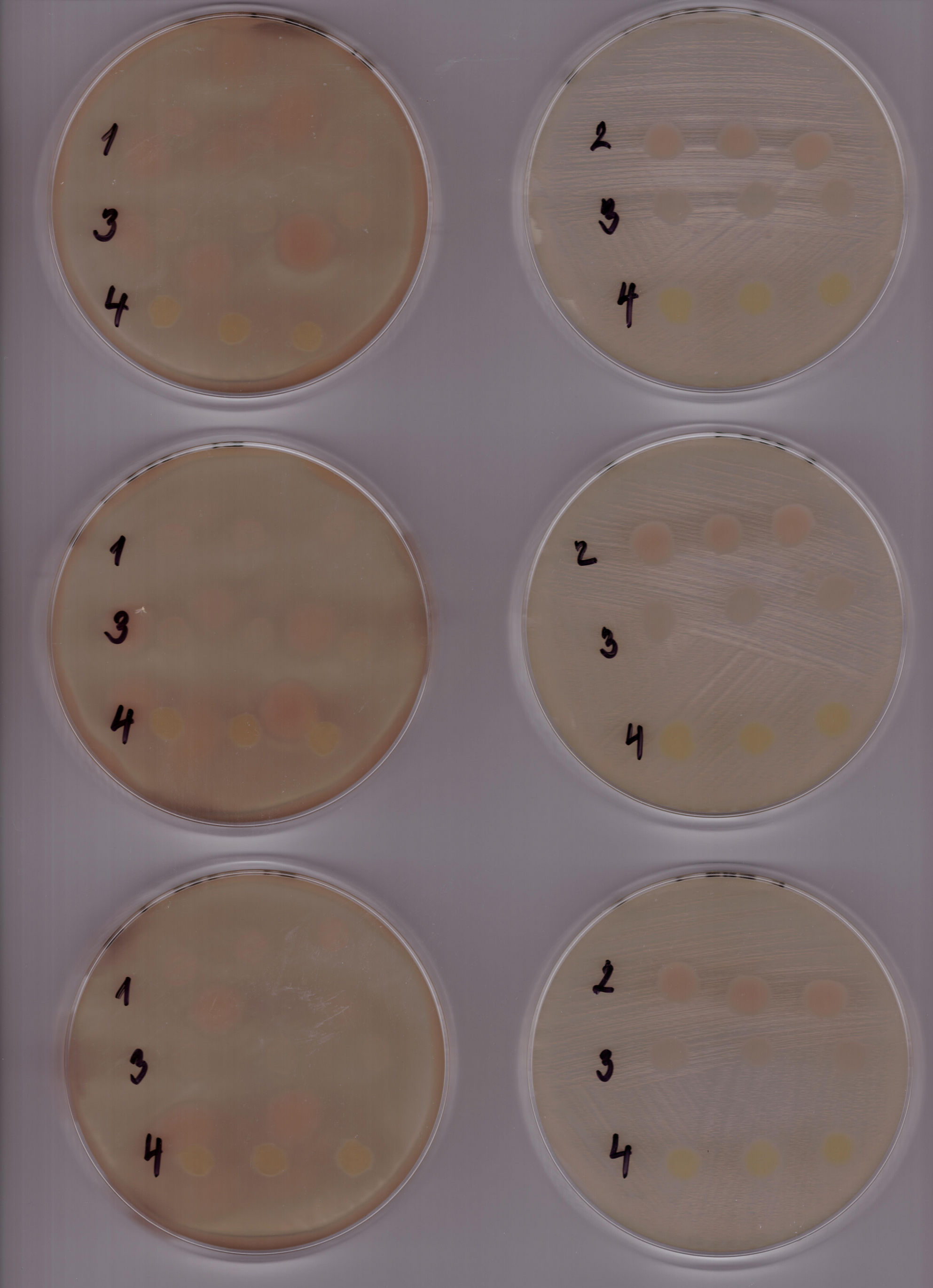

Supplement: Supplementary file 4 — Supplementary Figure 3a. [file 41598_2021_81937_MOESM4_ESM.tiff]

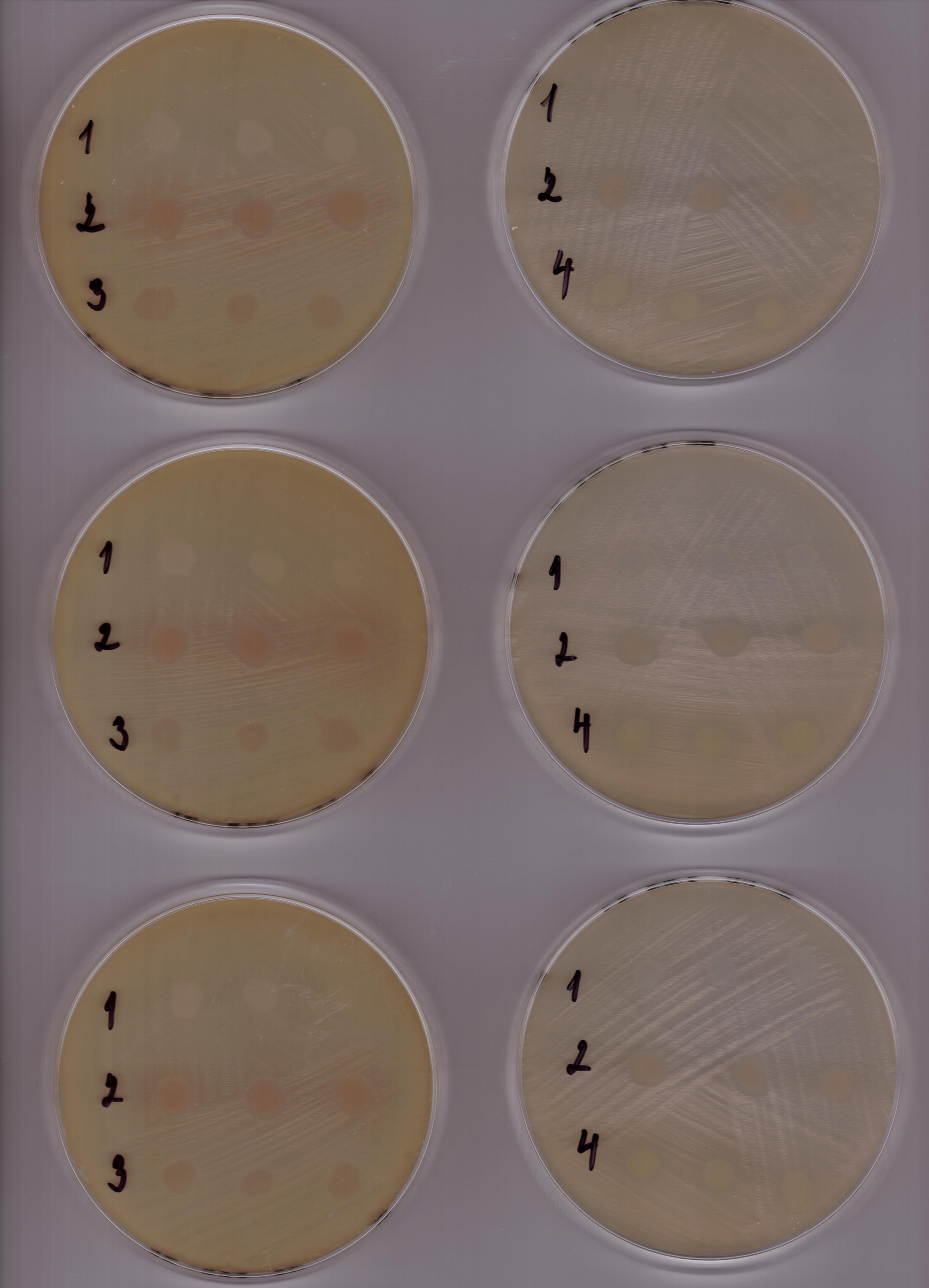

Supplement: Supplementary file 5 — Supplementary Figure 3b. [file 41598_2021_81937_MOESM5_ESM.tiff]
