# Supplementary material for: Bacterial and fungal endophyte communities in healthy and diseased oilseed rape and their potential for biocontrol of Sclerotinia and Phoma disease
Source: Sci Rep. 2021 Feb 15;11:3810. doi: 10.1038/s41598-021-81937-7 (PMC7884388; doi:10.1038/s41598-021-81937-7)
Supplement: Supplementary file 7 — Supplementary Information 2. [file 41598_2021_81937_MOESM7_ESM.pdf]

Supplementary Tables

For:

Bacterial and fungal endophyte communities in healthy and diseased oilseed rape and their potential for biocontrol of *Sclerotinia* and *Phoma* Disease

Authors: [1]Schmidt C.S., [1]*Mrnka L., [2]Lovecká P., [1]Frantík T., [2]Fenclová M., [2]Demnerová K., [1]Vosátka M.

*corresponding author (e-mail: libor.mrnka@ibot.cas.cz, tel.: +420271015311)

[1]*Institute of Botany of the CAS, Department of Mycorrhizal Symbioses, Průhonice - Chotobuz –, Czech Republic;*

[2]*University of Chemistry and Technology, Prague, Czech Republic*

Table S1: List of bacterial endophytes isolated from ripe oilseed rape and their plant growth-promoting *in vitro* traits; Genera are colour-coded as in Fig. 4 of the main article (PCA-analysis)

							Anti-Oxidative Activity			Phytohormones [ng ml^{-1}]		
Group	Isolate [a)]	Species	MALDI Score	SID [b)]	P-SOL [c)]	NH_3	AA [mg L^{-1}]	AOQ [%]	ACC-d	iP	iPR	IAA
Sphingobacterium	SP_RH21	*S. faecium*	1.848	-	-	+	17.9	76.1	-	1.43	3.8	6.75
Achromobacter	**AP_RD9**	***A. piechaudii***	**2.153**	**+**	**+**	**+**	**14.8**	**62.9**	**+**	**< LOQ**	**1.6**	**28.85**
Pseudomonas corrugata Clade [f)]	**PB_RH2**	***P. brassicacearum***	**2.313**	**+**	**+**	**++**	**18**	**76.5**	**+**	**4.69**	**< LOQ**	**13.55**
	**PB_RH20**	***P. brassicacearum***	**2.154**	**+**	**++**	**+**	**8.1**	**34.7**	**+**	**1.9**	**n.d.**	**10.39**
	PB_RH15	*P. brassicacearum*	2.369	++	-	+	/*	/*	-	/*	/*	/*
	PB_RH13	*P. brassicacearum*	2.088	-	-	+	/*	/*	-	/*	/*	/*
	PB_RH12	*P. brassicacearum*	2.257	+	/	-	/*	/*	-	/*	/*	/*
	PB_RH23	*P. brassicacearum*	2.171	-	/	-	13.9	59.1	-	2.24	n.d.	9.71
	**PKi_RH9**	***P. kilonensis***	**2.244**	**++**	**++**	**++**	**6.7**	**28.8**	**+**	**1.83**	**n.d.**	**12.14**
	PTh_RH3w	*P. thivervalensis*	2.005	-	-	+	10.3	44	-	1.75	n.d.	16.37
	PTh_RH3v	*P. thivervalensis*	1.991	-	-	+	/*	/*	-	/*	/*	/*
	50 ŘKK 21	*P. thivervalensis*	2.051	+	/	-	15.4	65.6	-	2.13	n.d.	22.05
Pseudomonas koreensis clade	PK_RD19A	*P. koreensis*	2.127	++	-	+	13.3	56.8		10.09	3.66	11.51
Pseudomonas proteolytica clade	PP_RD7	*P. proteolytica*	2.256	-	/	-	17.6	75	-	2.12	n.d.	< LOQ
Pseudomonas flourescens clade	PTo_RD16	*P. antarctica*	2.137	++	+	++	17.8	75.8	-	0.51	< LOQ	38.75
	PA_SD1	*P. antarctica*	2.186	+	-	+	10.3	44	-	2.54	n.d.	24.57
	PA_RD10B	*P. antarctica*	2.193	+	-	-	19.5	82.9	-	3.92	n.d.	41.96
	PA_RD13	*P. antarctica*	2.298	-	/	-	15.2	64.9	-	2.4	n.d.	< LOQ
	PE_RH22	*P. extremorientalis*	2.155	++	+	++	9.8	41.9	-	1.95	n.d.	9.91
	[d)] **PE_RD11**	***P. extremorientalis***	**2.073**	**++**	**++**	**+**	**16.8**	**71.6**	**+**	**3.44**	**n.d.**	**65.81**
	PE_RD41	*P. extremorientalis*	2.288	++	++	+	17.1	72.6	-	4.13	n.d.	53.67
	PE_RD3w	*P. extremorientalis*	2.264	++	+	+	17.8	75.7	-	2.16	n.d.	19.47
	PE_RD3v	*P. extremorientalis*	2.156	++	/	-	14.7	62.5	-	2.42	n.d.	18.89
	PE_RD15	*P. extremorientalis*	2.321	++	-	-	17.9	76.4	-	3.5	n.d.	23.8
	PF_RD1B	*P. fluorescens*	2.159	+	-	+	14.3	61.1	-	4.98	n.d.	10.68

Table S1, continued

Group	Isolate [a)]	Species	MALDI Score	SID [b)]	P-SOL [c)]	NH_3	Anti-Oxidative Activity AA [mg L^{-1}]	AOQ [%]	ACC-d	Phytohormones [ng ml^{-1}] iP	iPR	IAA
	**PG_RD39**	***P. grimontii/fluorescens*** [g)]	**2.104**	**++**	**+**	**+**	**18.3**	**77.9**	**+**	**6.14**	**n.d.**	**8.55**
	PG_RD6	*P. grimontii*	2.078	+	/	-	17.2	73.1	-	2.11	n.d.	8.98
	PG_RD14	*P. grimontii*	2.096	-	/	-	17.9	76.1	-	3.14	n.d.	17.39
	**PA_RD1A**	***P. antarctica/libanensis*** [g)]	**2.257**	**+**	**+**	**+**	**15.1**	**64.3**	**+**	**2.15**	**n.d.**	**11.22**
	PL_RD12B	*P. libanensis*	2.157	++	/	-	12.5	53.4	-	11.34	n.d.	7.77
	PR_RD17	*P. rhodesiae*	2.166	++	+	+	12.6	53.5	-	2.67	n.d.	< LOQ
	**PS_RH25**	***P. synxantha***	**2.143**	**+**	**+(72h)**	**+**	**16.8**	**71.7**	**+**	**8.36**	**n.d.**	**14.33**
	PS_RH8	*P. synxantha*	2.256	+	-	+	/*	/*	-	/*	/*	/*
	PTo_RH31	*P. tolaasii*	2.116	++	/	-	13.9	59.3	-	10.62	n.d.	12.87
	PTo_RH30	*P. tolaasii*	2.168	++	/	-	10.7	45.7	-	1.91	n.d.	12.33
	PTo_RH27	*P. tolaasii*	2.093	++	-	-	9.9	42.2	-	2.03	n.d.	11.07
	PTo_RH9	*P. tolaasii*	2.086	+	/	-	/*	/*	-	/*	/*	/*
	PTo_RD28C	*P. tolaasii*	2.243	++	+	+	4.4	19	-	10.93	< LOQ	14.42
	Pto_RD16	*P. tolaasii*	2.107	++	/	-	18.1	76.8	-	3.65	n.d.	20.49
	**PVe_RD1**	***P. veronii***	**2.134**	**++**	**++**	**+**	**14.1**	**60.2**	**+**	**2.37**	**n.d.**	**28.17**
Other	**PCh_RH1**	***P. chlororaphis/veronii*** [g)]	**2.143**	**-**	**++**	**+**	**16.9**	**72.1**	**+**	**3.2**	**n.d.**	**19.04**
Pseudomonas	PVi_SD24	*P. viridiflava*	1.95	+	++	+	17	72.2	-	5.73	n.d.	30.98
	PVi_SD4	*P. viridiflava*	2.161	-		+	15.5	65.9	-	2.8	n.d.	11.95
Steno-	SS_RD24	*S. rhizophila*	1.929	-	-	+	14.8	63.1	+	6.44	< LOQ	8.64
tropho-	SS_RD8	*Stenotrophomonas sp.*	2.27	+	/	-	15.3	65	-	24.01	n.d.	22.39
monas	SS_RD7	*Stenotrophomonas sp.*	2.346	+	-	-	14.2	60.4	-	24.53	n.d.	20.44
	SS_SH8	*Stenotrophomonas sp.*	2.007	-	/	-	14.9	63.5	-	4.05	0.71	16.75
	**SS_RD24**	***Stenotrophomonas sp.***	**2.428**	**-**	**/**	**-**	**13.8**	**59**	**-**	**23.7**	**n.d.**	**16.9**
	SS_RD31A	*Stenotrophomonas sp.*	2.335	-	/	-	18.5	78.5	-	2.38	0.7	44.53
	SS_RD42	*Stenotrophomonas sp.*	2.264	-	/	-	15.5	66.2	-	n.d.	n.d.	11.9
	SS_SH9A	*Stenotrophomonas sp.*	2.237	+	+	-	15.2	64.7	-	1.77	< LOQ	11.9
Enterobacter	EA_RH4w	*E. amnigenus*	2.235	-	/	-	/*	/*	-	/*	/*	/*
	EA_RH5	*E. amnigenus*	2.084	+	/	-	/*	/*	-	/*	/*	/*
	EA_RH_6v	*E. amnigenus*	2.121	-	/	-	/*	/*	-	/*	/*	/*

Table S1, continued

Group	Isolate [a)]	Species	MALDI Score	SID [b)]	P-SOL [c)]	NH_3	Anti-Oxidative Activity AA [mg L^{-1}]	Anti-Oxidative Activity AOQ [%]	ACC-d	Phytohormones [ng ml^{-1}] iP	Phytohormones [ng ml^{-1}] iPR	Phytohormones [ng ml^{-1}] IAA
Enterobacter, continued	EA_RH1	*E. amnigenus*	2.275	-	+	++	/*	/*	-	/*	/*	/*
	EA_RH2	*E. amnigenus*	2.206	-	/	-	/*	/*	-	/*	/*	/*
	EA_RH4v	*E. amnigenus*	2.172	+	/	-	10.1	43.2	-	4.43	< LOQ	< LOQ
	EA_RH6w	*E. amnigenus*	2.096	+	/	-	/*	/*	-	/*	/*	/*
	EA_RH7	*E. amnigenus*	2.244	-	/	-	/*	/*	-	/*	/*	/*
	EA_RH8	*E. amnigenus*	2.117	-	/	-	8.1	34.6	-	5.33	n.d.	6.65
	EA_RH10	*E. amnigenus*	2.326	++	-	-	9.1	38.9	-	6.64	< LOQ	7.33
	EA_RH11	*E. amnigenus*	2.067	-	/	-	/*	/*	-	/*	/*	/*
	EA_RH14	*E. amnigenus*	2.397	-	/	-	/*	/*	-	/*	/*	/*
	EA_RH15	*E. amnigenus*	2.387	++	+(72h)	+	10.4	44.2	-	4.87	< LOQ	< LOQ
	EA_RH17	*E. amnigenus*	2.276	-	+	++	16.3	69.3	-	3.82	< LOQ	36.18
	**EA_RH18**	***E. amnigenus***	**2.447**	**++**	**+(72h)**	**-**	**5.9**	**25.3**	**+**	**4.1**	**< LOQ**	**9.13**
	**EA_RH26**	***E. amnigenus***	**2.365**	**++**	**+**	**+**	**16.5**	**70.1**	**+**	**8.13**	**n.d.**	**27.1**
	EA_RD28B	*E. amnigenus*	2.267	-	/	-	19.1	81.2	-	n.d.	< LOQ	5.73
	EA_SH3	*E. amnigenus*	2.351	+	/	-	9.6	40.9	-	10.72	< LOQ	15.25
Erwinia	EP_RD11	*E. persicina*	2.162	-	-	+	16.4	69.7	-	2.78	< LOQ	10.93
	EP_RD9	*E. persicina*	2.328	-	/	-	17.9	76.2	-	2.58	< LOQ	10
	EP_RD10	*E. persicina*	2.225	-	/	-	17.4	74.1	-	9.22	< LOQ	11.65
	EP_RD12A	*E. persicina*	2.293	-	/	-	16	68.3	-	15.29	0.3	10.73
	EP_RD31B	*E. pescina*	2.105	-	/	-	18.6	79.3	-	9.08	< LOQ	18.31
Pectobacterium	PC_RD5	*P. carotovorum*	1.907	-	-	-	19.5	82.9	-	3.15	< LOQ	16.37
Serratia	**SP_RH21**	***S. proteamaculans***	**2.379**	**++**	**++**	**+**	**15.7**	**66.8**	**+**	**5.54**	**< LOQ**	**12.77**
	**SP_SH2**	***S. proteamaculans***	**2.346**	**-**	**+**	**+**	**8.7**	**37.3**	**+**	**1.91**	**0.48**	**< LOQ**
	**SP_SH13**	***S. proteamaculans***	**2.305**	**++**	**+**	**+**	**15.5**	**65.8**	**+**	**9.87**	**n.d.**	**6.46**
	**SP_RD8**	***S. proteamaculans***	**2.412**	**++**	**+**	**+**	**14.4**	**61.3**	**+**	**4.04**	**< LOQ**	**18.21**
	SP_RD37B	*S. proteamaculans*	2.06	++	-	+	15.7	66.8	-	7.79	2.7	8.84
	**SP_RD10A**	***S. proteamaculans***	**2.41**	**++**	**+**	**+**	**19**	**80.7**	**+**	**1.05**	**0.99**	**48.81**
	SP_RD12	*S. proteamaculans*	2.271	++	/	-	19.1	81.4	-	2.7	< LOQ	38.85
	SP_RD19B	*S. proteamaculans*	2.039	+	/	-	15.5	66	-	2.91	n.d.	12.53

Table S1, continued

Group	Isolate [a)]	Species	MALDI Score	SID [b)]	P-SOL [c)]	NH_3	Anti-Oxidative Activity: AA [mg L^{-1}]	Anti-Oxidative Activity: AOQ [%]	ACC-d	Phytohormones [ng ml^{-1}]: iP	Phytohormones [ng ml^{-1}]: iPR	Phytohormones [ng ml^{-1}]: IAA
Serratia, continued	SP_RD25	*S. proteamaculans*	2.318	+	/	-	17.3	73.6	-	< LOQ	n.d.	87.66
	SP_RD38B	*S. proteamaculans*	2.354	+	/	-	18.2	77.2	-	2.8	0.71	53.67
	SP_RD43	*S. proteamaculans*	2.385	+	/	-	14.9	63.7	-	0.54	< LOQ	58.04
	SP_RD26	*S. proteamaculans*	2.239	-	/	-	17.8	75.6	-	4.02	n.d.	28.51
	SP_RD36B	*S. proteamaculans*	2.306	-	/	-	13.5	57.5	-	0.89	n.d.	74.55
	SP_RD38C	*S. proteamaculans*	2.381	-	/	-	18.9	80.4	-	2.34	< LOQ	53.67
	SP_SD28B	*S. proteamaculans*	2.267	-	/	-	14.5	61.9	-	1.96	< LOQ	53.18
	SP_RD38A	*S. proteamaculans*	2.281	+	/	-	18.6	79.3	-	2.69	< LOQ	64.84
	SP_SD2	*S. proteamaculans*	2.298	+	/	-	16.9	72.1	-	< LOQ	n.d.	43.71
	SP_SD7	*S. proteamaculans*	2.431	+	/	-	18.5	78.6	-	1.07	n.d.	39.77
	SP_SD11	*S. proteamaculans*	2.296	+	/	-	19.1	81.2	-	< LOQ	< LOQ	33.36
	SP_SD12A	*S. proteamaculans*	2.348	+	/	-	18.6	79.3	-	2.72	n.d.	30.16
	SP_SD21A	*S. proteamaculans*	2.188	+	/	-	18.6	79	-	7.97	n.d.	28.8
	SP_SD21C	*S. proteamaculans*	2.444	+	/	-	15.9	67.6	-	3.62	< LOQ	16.8
Bacillus	BC_RH29	*B. cereus*	2.301	+	/	-	13.8	58.7	-	2.62	n.d.	10.2
	BC_SH6v	*B. cereus*	2.204	+	/	-	15.9	67.7	-	5.94	< LOQ	7.53
	BC_SH1	*B. cereus*	2.359	-	/	-	7.5	32.3	-	1.72	0.5	< LOQ
	BC_SH6w	*B. cereus*	2.100	-	/	-	9.8	41.9	-	1.37	0.39	13.45
	BC_SH10	*B. cereus*	2.153	-	/	-	15.4	65.5	-	9.41	< LOQ	< LOQ
	BC_SD23	*B. cereus*	2.368	-	/	-	14	59.7	-	12.59	< LOQ	12.33
	BC_SD26	*B. cereus*	2.166	-	/	-	9.8	42	-	10.72	< LOQ	19.86
	BM_RD4	*B. mycoides*	2.175	++	-	-	16.3	69.6	-	10.41	n.d.	11.9
	BS_SD10	*B. subtilis*	1.864	-	/	-	8.8	37.7	-	7.52	1.05	6.36
Staphylo-coccus	SC_SD17	*S. capitis*	2.169	-	-	+	15.8	67.3	-	2.85	< LOQ	40.7
	SW_RH19	*S. warneri*	1.991	+	/	++	<LOQ	-	-	2.14	0.52	19.72
	SS_RD7	*S. warneri*	1.752	+	/	-	/*	/*	-	/*	/*	/*
	StP_SD13	*S. pasteuri*	2.023	+	-	-	17.6	75.1	-	< LOQ	n.d.	43.13
No ID	No_SD19	No ID	/	/	/	/	/	/	/	/	/	/

[a] RH = isolate from roots of healty plants; SH = isolate from shoots of healty plants; RD = isolate from roots of diseased plants, SD = isolate from shoots of diseased plants

[b] SID, Siderophore production; P-SOL, P-solubilisation; AA, Antioxidative-activity expressed as ascorbic acid equivalent; AOQ, total antioxidative activity expressed in % of quenching activity; ACC-d, ACC-d 1-1-aminocyclopropane-1-carboxylate deaminase activity; iP, N6-(Δ2-isopentenyl)adenine; iPR, N6-(Δ2-isopentenyl)adenosine; IAA, indole-3-acetic acid

[c] ++ = strong production; + production; - = no production, n.d. = not detected; < LOQ below quantitation limit; / = not measured; /* = not measured because isolate did not grow in liquid medium

[d] Isolates in bold: chosen for follow-on experiments with oilseed rape

[f] Clades of *Pseudomonas* according to Garrido-Sanz D, Meier-Kolthoff JP, Göker M, Martín M, Rivilla R, Redondo-Nieto M (2016) Genomic and Genetic Diversity within the *Pseudomonas fluorescens* Complex. PLoS ONE 11 (2): e0150183. doi:10.1371/journal.pone.0150183

[g] repeated MALDI-TOF runs gave conflicting results: both species names are listed

Table S2: List of fungal endophytes isolated from ripe oilseed rape and their plant growth-promoting *in vitro* traits; Genera are colour-coded as in Fig. 4 of the main article (PCA-analysis)

						Anti-Oxidative Activity		Phytohormones [ng ml^{-1}]			Mycotoxins	
	Class	Order	Family	Genus	Isolate	AA [mg L^{-1}]	AOQ [%]	iP	iPR	IAA	Roquefortin C	Meleagrin
Asco-my-cetes	*Dothideo-mycetes*	*Capno-diales*	*Cladosporia-ceae*	*Cladosporium*	RHR6	23,69	19,10	3,71	0,35	490,40	/	/
				Cladosporium	**RHR8**	**22,49**	**17,96**	**10,48**	**0,73**	**111,47**	**n.d.**	**n.d.**
				Cladosporium	**RHR9**	**92,39**	**84,62**	**4,21**	**10,34**	**712,73**	**n.d.**	**n.d.**
				Cladosporium	**RHR7.**	**33,51**	**28,47**	**2,79**	**n.d.**	**881,38**	**n.d.**	**n.d.**
				Cladosporium	**RHR14**	**70,86**	**64,08**	**< 0,5**	**13,42**	**61,83**	**/**	**/**
				Cladosporium	**RHR17**	**31,18**	**26,25**	**10,85**	**n.d.**	**237,78**	**n.d.**	**n.d.**
				Cladosporium	**RHS5**	**27,85**	**23,06**	**4,49**	**n.d.**	**355,24**	**n.d.**	**n.d.**
				Cladosporium	**RHS17**	**26,90**	**22,16**	**8,53**	**n.d.**	**109,06**	**n.d.**	**n.d.**
	Dothideo-mycetes	Capno-diales	*Mycosphaer-ellaceae*	*Related to Periconiella*	RHR11	89,94	82,28	< 0,5	23,44	315,48	n.d.	n.d.
	Dothideo-mycetes	Capno-diales	*Mycosphaer-ellaceae*	*Ramularia*	RDS14	20,86	16,40	8,93	< 0,25	210,43	n.d.	n.d.
	Dothideo-mycetes	*Pleo-sporales*	*Didymella-ceae*	*Leptosphaerulina*	RHR16	28,35	23,54	n.d.	10,03	29,89	n.d.	n.d.
	Dothideo-mycetes	*Pleo-sporales*	*Lepto-sphaeria-ceae*	*Leptosphaeria*	RHS4	41,01	35,62	< 0,5	10,42	139,63	n.d.	n.d.
				Leptosphaeria	RHS8	20,10	15,68	n.d.	8,09	17,02	n.d.	n.d.
				Leptosphaeria	RDS9	30,74	25,83	< 0,5	9,40	234,56	/	/
	Dothideo-mycetes	*Pleo-sporales*	*Didymella-ceae*	*Epicoccum*	RHR2	47,12	41,44	n.d.	15,63	308,58	/	/
				Epicoccum	RHR1	35,59	30,45	n.d.	18,30	740,59	n.d.	n.d.
				Epicoccum	RHR15	/	/	/	/	/	< 125	< 125
	Dothideo-mycetes	*Pleo-sporales*	*Torulaceae*	*Torula*	RHR3	93,34	85,53	< 0,5	20,86	808,73	n.d.	n.d.
	Dothideomycetes incerta sedis			*Peltaster*	RDS15	90,25	82,58	0,50	12,65	53,59	n.d.	n.d.
	Eurotio-mycetes	*Euro-tiales*	*Asper-gillaceae*	*Penicillium chrysogenum*	RHR13	31,56	26,61	< 0,5	18,63	117,91	32 723	47 891

Table S2, continued

	Class	Order	Family	Genus	Isolate	Anti-Oxidative Activity AA [mg L^{-1}]	AOQ [%]	Phytohormones [ng ml^{-1}] iP	iPR	IAA	Mycotoxins Roque-fortin C	Melea-grin
Asco-my-cetes	*Sordario-mycetes*	*Glomere-llales*	*Plecto-sphaerellaceae*	*Plectosphaerella*	RHR12	98,94	90,87	n.d.	0,41	3 003,22	n.d.	n.d.
				Plectosphaerella	RDR9	56,75	50,63	n.d.	5,16	146,87	n.d.	n.d.
				Plectosphaerella / Monographella	RDR3	45,42	39,82	< 0,5	5,34	889,43	n.d.	n.d.
				Plectosphaerella	RHS2	38,36	33,09	n.d.	6,76	166,98	n.d.	n.d.
				Plectosphaerella/ Monographella	RDR7	35,09	29,97	n.d.	6,90	786,45	/	/
	Sordario-mycetes	*Hypo-creales*	*Nectriaceae*	*Fusarium*	RDR8	60,28	53,99	< 0,5	4,44	491,20	n.d.	n.d.
Basi-dio-my-cetes	*Agarico-mycetes*	*Cantharel-lales / Polypo-rales*	*Phanero-chaetaceae / Cerato-basidiaceae*	*Bjerkandera / Thanatephorus*	RDR10	32,82	27,81	< 0,5	14,93	773,58	n.d.	n.d.
	Tremello-mycetes	*Tremel-lales / Tricho-sporales*	*Bulleribasidiaceae/Trichosporaceae*	*Vishniacozyma / Cryptococcus*	RDS4	23,88	19,28	1,27	19,88	1 171,01	n.d.	n.d.
	Tremello-mycetes	*Holtermannia-les / Filo-basidiales*	*-/Filobasidiaceae*	*Holtermanniella / Cryptococcus*	RDS12	44,79	39,22	3,47	13,60	3 021,40		
				Holtermanniella / Cryptococcus	RDS8	26,84	22,10	0,94	8,75	435,69	n.d.	n.d.

[a] RHR = isolate from roots of healthy plants; RHS = isolate from shoots of healthy plants; RDR = isolate from roots of diseased plants, RDS = isolate from shoots of diseased plants [b] SID, Siderophore production; P-SOL, P-solubilisation; AA, Anitoxidative activity expressed as ascorbic acid equivalent; AOQ, total antioxidative activity expressed in % of quenching activity ; ACC-d, 1-1-aminocyclopropane-1-carboxylate deaminase activity; iP, N6-(Δ2-isopentenyl)adenine; iPR, N6-(Δ2-isopentenyl)adenosine; IAA, indole-3-acetic acid [c] n.d. = not detected; < LOQ below quantitation limit; / = not measured; /* = not measured because the isolate did not grow in liquid medium; Isolates in bold: chosen for follow-on experiments with oilseed rape

Table S3: Effects of bacterial endophytes on growth and pod yield of oilseed rape grown in phosphorus depleted substrate amended with apatite (11 g L^{-1}). Averages and SE: for growth stage, Averages (Range)

Treatment		Harvest Parameters [a)]								
	BBCH Growth Stage at 173d[b)]	No. of shoots		Shoot Height [cm]	Cumulative Shoot Height [cm]		Shoot DW [g]		Root DW [g]	
C [c)]	51.2 (30-59)	3.5 + 0.4	bc	146 + 8	233 + 18	b	12 + 0.4	bc	3.9 + 0.2	b
Cp [d)]	64.4 (63-65)	7.0 + 0.8	a***	139 + 18	452 + 52	a***	19 + 0.5	a***	6.4 + 8.1	a***
***Achromobacter piechaudii* AP_RD9 [e)]**	**59.6 (51-65)**	**4.0 + 0.5**	**bc**	**169 + 8**	**271 + 25**	**b**	**12 + 0.3**	**bc**	**3.8 + 9.2**	**b**
***Pseudomonas chlororaphis/veronii* PCh_RH1**	**56.4 (51-65)**	**3.2 + 0.4**	**bc**	**160 + 9**	**242 + 16**	**b**	**11 + 1.2**	**bc**	**4.1 + 0.5**	**b**
P. brassicacearum PB_RH2	57.8 (50-65)	4.2 + 0.7	bc	167 + 16	284 + 40	b	13 + 0.1	bc	4.3 + 3.3	b
P. brassicacearum PB_RH20	46.0 (30-60)	3.0 + 0.3	bc	158 + 12	221 + 34	b	13 + 0.9	bc	4.2 + 3.5	b
P. kilonensis PKi_RH9	43.0 (30-53)	2.4 + 0.6	bc	156 + 12	195 + 16	b	12 + 0.8	bc	4.2 + 3.4	b
P. synxantha PS_RH25	56.0 (52-59)	2.8 + 0.5	bc	177 + 8	241 + 19	b	13 + 0.4	bc	4.4 + 0.1	b
P. antarctica/libanenensis PA_RD1A	47.2 (30-52)	3.2 + 0.2	bc	161 + 8	211 + 13	b	12 + 0.6	bc	4.7 + 8.3	b
P. veronii PVe_RD1	50.2 (30-65)	2.0 + 0.4	c	142 + 16	176 + 34	b	12 + 1.1	bc	3.8 + 7.5	b
P. extremorientalis PE_RD11	51.6 (30-65)	4.0 + 0.7	bc	171 + 10	276 + 27	b	13 + 1.1	bc	4.4 + 8.5	b
***Pseudomonas sp.* PG_RD39**	**59.0 (53-65)**	**3.8 + 0.4**	**bc**	**163 + 9**	**294 + 28**	**b**	**12 + 0.5**	**bc**	**4.8 + 2.2**	**ab**
***Stenotrophomonas* SS_RD24**	**50.0 (30-61)**	**3.6 + 0.7**	**bc**	**144 + 7**	**236 + 24**	**b**	**13 + 1.0**	**bc**	**4.0 + 4.3**	**b**
Enterobacter amnigenus EA_RH18	53.2 (30-62)	4.8 + 0.7	ab	161 + 10	309 + 47	ab	14 + 1.3	b	4.4 + 5.6	b
E. amnigenus EA_RH26	45.8 (30-59)	3.2 + 0.2	bc	161 + 16	229 + 20	b	12 + 0.5	bc	4.5 + 9.4	b
Serratia proteamaculans SP_RH21	54.4 (51-57)	2.8 + 0.5	bc	160 + 6	257 + 35	b	10 + 0.9	c	3.7 + 5.4	b
S. proteoaccumulans SP_SH13	39.0 (30-55)	3.4 + 0.2	bc	143 + 17	196 + 26	b	13 + 0.3	bc	4.6 + 4.3	b
S. proteamaculans SP_SH2	53.2 (50-57)	3.8 + 0.7	bc	162 + 10	256 + 26	b	12 + 0.6	bc	4.1 + 6.3	b
S. proteamaculans SP_RD8	44.4 (30-55)	3.2 + 0.2	bc	165 + 8	238 + 25	b	13 + 0.3	bc	4.3 + 9.1	b
S. proteamaculans SP_RD10A	54.4 (51-65)	3.6 + 0.4	bc	153 + 12	253 + 39	b	13 + 1.1	bc	4.4 + 2.3	b

[a)] Means + SE; stars indicate difference to control C according Dunnet's test at $P < 0.05$ (*), $P < 0.01$ (**) and $P < 0.001$ (***); values followed by the same letters are not significantly different according to Unequal N HSD test; if no letters are shown, Unequal HSD test yielded no significant differences (except for the full phosphorus control (Cp),

[b)] BBCH growth stage: means (range)

[c)] Control, with 50 mg P per pot, as in treatments, [d)] Control with elevated phosphorus dose (260 mg P per pot),

[e)] Isolates selected for field experiment are shown in bold

Table S3 continued: Pod Yield

Treatment	Pod No. a)				Pod Dry Weight [g] a)			
	All		Mature		All		Mature	
C c)	45 + 4	b	25 + 6		0.5 + 0.11	b	0.5 + 0.12	b
Cp d)	116 + 16	a***	101 + 9	***	4.0 + 0.44	a***	3.9 + 0.45	a***
***Achromobacter piechaudii* AP_RD9 e)**	**53 + 11**	**b**	**40 + 12**		**1.7 + 0.55**	**b***	**1.7 + 0.55**	**b**
***Pseudomonas chlororaphis/veronii* PCh_RH1**	**58 + 12**	**b**	**47 + 15**		**1.6 + 0.59**	**b**	**1.6 + 0.60**	**b**
P. brassicacearum PB_RH2	40 + 11	b	18 + 5		0.4 + 0.12	b	0.3 + 0.13	b
P. brassicacearum PB_RH20	42 + 11	b	18 + 8		0.5 + 0.21	b	0.4 + 0.22	b
P. kilonensis PKi_RH9	48 + 10	b	32 + 11		0.7 + 0.29	b	0.7 + 0.29	b
P. synxantha PS_RH25	52 + 8	b	32 + 10		0.8 + 0.25	b	0.7 + 0.26	b
P. antarctica/libanenensis PA_RD1A	40 + 9	b	27 + 8		0.7 + 0.23	b	0.6 + 0.23	b
P. veronii PVe_RD1	46 + 13	b	32 + 8		1.3 + 0.37	b	1.2 + 0.35	b
P. extremorientalis PE_RD11	49 + 6	b	27 + 7		0.8 + 0.26	B	0.7 + 0.28	b
***Pseudomonas sp.* PG_RD39**	**57 + 13**	**b**	**31 + 7**		**0.7 + 0.14**	**b**	**0.7 + 0.13**	**b**
***Stenotrophomonas* SS_RD24**	**51 + 13**	**b**	**35 + 12**		**1.2 + 0.48**	**b**	**1.2 + 0.48**	**b**
Enterobacter amnigenus EA_RH18	62 + 12	b	46 + 10		1.4 + 0.34	b	1.3 + 0.33	b
E. amnigenus EA_RH26	50 + 12	b	31 + 11		0.8 + 0.32	b	0.7 + 0.32	b
Serratia proteamaculans SP_RH21	68 + 6	ab	42 + 9		1.1 + 0.27	b	1.0 + 0.29	b
S. proteoaccumulans SP_SH13	34 + 8	b	11 + 9		0.3 + 0.22	b	0.3 + 0.23	b
S. proteamaculans SP_SH2	66 + 7	ab	23 + 11		0.6 + 0.29	b	0.5 + 0.30	b
S. proteamaculans SP_RD8	47 + 12	b	26 + 9		0.5 + 0.18	b	0.4 + 0.17	b
S. proteamaculans SP_RD10A	34 + 12	b	18 + 12		0.4 + 0.24	b	0.4 + 0.24	b

a) Means + SE; stars indicate difference to control C according Dunnet's test at P < 0.05 (*), P < 0.01 (**) and P < 0.001 (***); values followed by the same letters are not significantly different according to Unequal N HSD test; if no letters are shown, Unequal HSD test yielded no significant differences (except for the high phosphorus control Cp)

b) Control, with 50 mg P per pot, as in treatments

c) Control with elevated phosphorus dose (260 mg P per pot)

d) Isolates selected for field experiment are shown in bold

Table S4: Effects of fungal endophytes on growth and pod yield of oilseed rape grown in phosphorus depleted substrate amended with apatite (11 g L^{-1}). Averages and SE, for growth stage, Averages (Range). Apart from Shoot DW, data were log-transformed to achieve normal distribution of residuals

Phylum	Class	Genus	Isolate	BBCH Growth Stage [b)] 18/5/2015 (166d growth)	184d growth	No. of Stems		Height [cm]		Cumulative Height [cm]		Shoot DW [g]		Root DW [g]	
		Control	C [c)]	57 (35-65)	71 (62-75)	2.9 ± 0.46	ab	143 ± 9	a	211 ± 8	b	11 ± 0.5	ab	4.1 ± 0.2	ab
		Cp	Cp [d)]	59 (35-65)	76 (74-77)	5.4 ± 0.51	a*	150 ± 5	a	400 ± 13	a*	15 ± 1.4	a*	5.6 ± 0.8	a
Asco-	*Dothi-*	*Cladosporium*	RHR7	51 (35-65)	69 (65-73)	2.6 ± 0.24	ab	162 ± 6	a	230 ± 22	b	12 ± 1.2	ab	3.9 ± 0.5	ab
mycota	*deo-*	*Cladosporium*	RHR8	57 (35-65)	71 (64-74)	3.0 ± 0.32	ab	158 ± 4	a	254 ± 28	ab	12 ± 0.7	ab	4.4 ± 0.3	ab
	mycetes	*Cladosporium*	RHR9	49 (35-65)	68 (63-72)	1.8 ± 0.20	b	148 ± 8	a	175 ± 12	b	11 ± 0.9	ab	3.9 ± 0.3	ab
		Cladosporium	RHS5	63 (55-65)	73 (71-73)	2.2 ± 0.37	ab	154 ± 5	a	181 ± 6	b	9 ± 0.8	b	3.3 ± 0.2	b
		Cladosporium	RHS17	57 (55-65)	71 (69-75)	3.0 ± 0.32	ab	141 ± 9	a	213 ± 15	b	10 ± 0.9	ab	3.6 ± 0.1	ab
		Ramularia	RDS14	55 (35-65)	71 (65-74)	2.4 ± 0.40	ab	162 ± 5	a	219 ± 25	b	11 ± 1.1	ab	4.0 ± 0.1	ab
		Related to Periconiella/Penidiella	RHR11	57 (35-65)	73 (67-76)	2.0 ± 0.32	b	146 ± 4	a	179 ± 7	b	10 ± 0.8	ab	3.9 ± 0.4	ab
		Epicoccum	RHR1	55 (35-65)	70 (67-75)	2.2 ± 0.49	b	151 ± 8	a	219 ± 28	b	12 ± 1.8	ab	3.7 ± 0.5	ab
		Epicoccum	RHR15	59 (55-65)	72 (71-73)	1.8 ± 0.20	b	162 ± 3	a	189 ± 10	b	11 ± 0.8	ab	4.2 ± 0.2	ab
		Torula	RHR3	51 (35-65)	70 (67-74)	2.6 ± 0.24	ab	155 ± 5	a	200 ± 15	b	11 ± 1.2	ab	4.4 ± 0.7	ab
		Leptosphaeria	RHS4	53 (35-65)	70 (65-73)	3.6 ± 1.12	ab	153 ± 5	a	272 ± 65	ab	10 ± 0.8	ab	3.8 ± 0.3	ab
	Sorda-	*Plectosphaerella*	RHR12	43 (35-65)	65 (53-71)	2.2 ± 0.58	b	137 ± 7	a	171 ± 15	b	11 ± 0.8	ab	3.4 ± 0.2	ab
	rio-	*Plectosphaerella*	RHS2	59 (55-65)	72 (69-75)	1.8 ± 0.37	b	155 ± 6	a	195 ± 22	b	10 ± 0.7	ab	4.0 ± 0.3	ab
	mycetes	*Plectosphaerella/ Monographella*	RDR3	57 (35-65)	71 (65-75	2.4 ± 0.40	ab	152 ± 6	a	195 ± 14	b	11 ± 0.9	ab	4.1 ± 0.4	ab
		Plectosphaerella	RDR9	57 (55-65)	68 (67-71)	2.6 ± 0.51	ab	160 ± 7	a	227 ± 22	b	10 ± 0.7	ab	3.5 ± 0.2	ab
	Eurotio-mycetes	*Penicillium chrysogenum complex*	RHR13	59 (55-65)	72 (71-74)	2.4 ± 0.24	ab	152 ± 9	a	195 ± 16	b	11 ± 0.8	ab	3.6 ± 0.4	ab
Basidio-	*Tremel-*	*Holtermanniella/ Cryptococcus*	RDS8	53 (35-65)	69 (65-72)	1.8 ± 0.20	b	163 ± 6	a	184 ± 9	b	11 ± 1.2	ab	3.5 ± 0.2	ab
mycota	*lomycetes*	*Vishniacozyma/ Cryptococcus*	RDS4	43 (35-55)	68 (65-73)	2.0 ± 0.32	b	156 ± 8	a	184 ± 16	b	12 ± 1.1	ab	4.0 ± 0.3	ab
	Agari-mycetes	*Piriformospora*	PIND	59 (35-65)	69 (53-74)	1.8 ± 0.37	b	148 ± 10	a	165 ± 12	b	11 ± 1.3	ab	3.9 ± 0.4	ab

Table S4 continued: Pod Yield

Phylum	Class	Order	Genus	Isolate	No. of Pods				Dry weight of pods [g]			
					All		Mature		All		Mature	
				C [c)]	49 ± 5	a	45 ± 6	a	2.5 ± 0.46	a	2.5 ± 0.46	a
				Cp [d)]	144 ± 11	b***	140 ± 13	b***	9.0 ± 0.83	b***	9.0 ± 0.86	b***
Asco-mycota	*Dothi-deo-mycetes*	*Capno-diales*	*Cladosporium*	RHR7	58 ± 8	a	47 ± 9	a	2.3 ± 0.60	a	2.3 ± 0.61	a
			Cladosporium	RHR8	47 ± 4	a	42 ± 4	a	2.6 ± 0.51	a	2.5 ± 0.54	a
			Cladosporium	RHR9	45 ± 7	a	36 ± 9	a	1.8 ± 0.73	a	1.7 ± 0.74	a
			Cladosporium	RHS5	52 ± 5	a	52 ± 5	a	3.4 ± 0.08	a	3.4 ± 0.08	a
			Cladosporium	RHS17	48 ± 4	a	46 ± 4	a	3.1 ± 0.36	a	3.1 ± 0.37	a
			Ramularia	RDS14	38 ± 6	a	36 ± 6	a	2.3 ± 0.65	a	2.3 ± 0.65	a
			Related to Periconiella/Penidiella	RHR11	48 ± 6	a	48 ± 6	a	3.3 ± 0.70	a	3.3 ± 0.69	a
		Pleo-sporales	*Epicoccum*	RHR1	66 ± 9	a	58 ± 4	a	2.9 ± 0.59	a	2.9 ± 0.60	a
			Epicoccum	RHR15	52 ± 4	a	51 ± 4	a	3.4 ± 0.27	a	3.4 ± 0.27	a
			Torula	RHR3	65 ± 10	a	57 ± 11	a	2.8 ± 0.86	a	2.7 ± 0.87	a
			Leptosphaeria	RHS4	56 ± 6	a	52 ± 6	a	2.7 ± 0.45	a	2.7 ± 0.45	a
	Sor-dario-mycetes	*Glomer-ellales*	*Plectosphaerella*	RHR12	41 ± 11	a	40 ± 11	a	1.6 ± 0.65	a	1.6 ± 0.66	a
			Plectosphaerella	RHS2	50 ± 4	a	43 ± 4	a	2.6 ± 0.44	a	2.6 ± 0.46	a
			Plectosphaerella/ Monographella	RDR3	48 ± 7	a	47 ± 7	a	2.9 ± 0.66	a	2.9 ± 0.66	a
			Plectosphaerella	RDR9	42 ± 8	a	38 ± 7	a	1.9 ± 0.43	a	1.9 ± 0.43	a
	Eurotio-mycetes	*Eurotiales*	*Penicillium chrysogenum complex*	RHR13	56 ± 6	a	53 ± 7	a	3.4 ± 0.69	a	3.4 ± 0.70	a
Basidio-mycota	*Tremello-mycetes*	*Holter-manniales/ Filobasidiales*	*Holtermanniella/Cryptococcus*	RDS8	59 ± 6	a	46 ± 6	a	2.2 ± 0.57	a	2.2 ± 0.59	a
		Tremellales / Trichosporales	*Vishniacozyma/Cryptococcus*	RDS4	48 ± 6	a	38 ± 7	a	1.8 ± 0.52	a	1.7 ± 0.54	a
	Agari-mycetes	*Sebacinales*	*Piriformospora*	PIND	52 ± 2	a	45 ± 9	a	2.8 ± 0.66	a	2.7 ± 0.69	a

Table S5: Effect of seed treatment with endophytes on oilseed rape emergence in *Sclerotinia* infested soil before onset of disease. Data are presented as means ± SE. C- = negative (healthy) control without *Sclerotinia* inoculation and without bacterial inoculation. C+ positive (pathogen-inoculated) control in *Sclerotinia* infested soil without bacterial inoculation. A. = *Achromobacter*, Ps. = *Pseudomonas*, E. = *Enterobacter*, S. = *Serratia.* There was a significant treatment effect on emergence (P < 0.0001). Treatments with the same letters are not significantly different according to Unequal N HSD test (inoculation with cell /bacteria/ or spore /RHR7/ suspension) or according to Kruskal-Wallis Test. Where Dunnett's test showed a significant treatment effect compared to the control C+ the significance of the treatment effect is shown as P value in superscript brackets. Isolates in bold were chosen for the follow-up field experiment with oilseed rape.

Isolate [a)]	Emergence	
	14 Days Growth	
Seed Treatment with Cell or Spore suspension		
Non-inoculated Control C-	*5.7 ± 0.33*	bc
Inoculated Control C+	*7.3 ± 0.33*	abc
***A. piechaudii* AP_RD9**	**6.7 ± 0.33**	**abc**
***Ps. chloraphis/veronii* PCh_RH1**	**7.5 ± 0.76**	**abc**
Ps. kilonensis PKi_RH9	8.7 ± 0.17	a
Ps. synxantha PS_RH25	8.2 ± 0.40	a
Ps. antarctica/libanenensis PA_RD1A	5.2 ± 0.48	c$^{(P=0.009)}$
Ps. veronii PVe_RD1	8.2 ± 0.40	a
Ps. extremorientalis PE_RD11	7.8 ± 0.48	ab
***Ps. grimontii/fluorescens* PG_RD39**	**6.3 ± 0.33**	**abc**
***Stenotrophomonas* sp. SS_RD24**	**7.7 ± 0.67**	**ab**
E. amnigenus EA_RH18	8.0 ± 0.37	ab
E. amnigenus EA_RH26	7.8 ± 0.79	ab
S. proteamaculans SP_RH21	7.5 ± 0.43	abc
S. proteamaculans SP_SH2	7.3 ± 0.21	abc
S. proteamaculans SP_RD8	7.0 ± 0.63	abc
S. proteamaculans SP_RD10A	8.0 ± 0.52	ab
Cladosporium RHR7 (Spores)	7.3 ± 0.49	abc
Mycelial Inoculation		
Non-inoculated Control C-	7.8 ± 0.48	ab
Inoculated Control C+	8.9 ± 0.23	a
Cladosporium RHR7	8.2 ± 0.31	ab

Cladosporium RHR8	8.3 ± 0.42	ab
Cladosporium RHR9	8.7 ± 0.21	ab
Cladosporium RHS5	8.0 ± 0.45	ab
Cladosporium RHS17	6.0 ± 0.63	b
